# Analyses of expressed sequence tags in *Neurospora* reveal rapid evolution of genes associated with the early stages of sexual reproduction in fungi

**DOI:** 10.1186/1471-2148-12-229

**Published:** 2012-11-27

**Authors:** Kristiina Nygren, Andreas Wallberg, Nicklas Samils, Jason E Stajich, Jeffrey P Townsend, Magnus Karlsson, Hanna Johannesson

**Affiliations:** 1 Department of Forest Mycology and Plant Pathology, Swedish University of Agricultural Sciences, Box 7026, SE-75007, Uppsala, Sweden; 2 Department of Medical Biochemistry and Microbiology, Uppsala University, Box 582, SE-751, Uppsala, Sweden; 3 Department of Plant Pathology and Microbiology, University of California at Riverside, 900 University Avenue, Riverside, CA, 92521, USA; 4 Department of Ecology and Evolutionary Biology, Yale University, 165 Prospect St., New Haven, CT, 06520-8106, USA; 5 Department of Evolutionary Biology, Evolutionary Biology Centre, Uppsala University, Norbyvägen 18 D, SE-752 36, Uppsala, Sweden

**Keywords:** *Neurospora*, Reproductive genes, dN/dS, Speciation, Microarray

## Abstract

**Background:**

The broadly accepted pattern of rapid evolution of reproductive genes is primarily based on studies of animal systems, although several examples of rapidly evolving genes involved in reproduction are found in diverse additional taxa. In fungi, genes involved in mate recognition have been found to evolve rapidly. However, the examples are too few to draw conclusions on a genome scale.

**Results:**

In this study, we performed microarray hybridizations between RNA from sexual and vegetative tissues of two strains of the heterothallic (self-sterile) filamentous ascomycete *Neurospora intermedia*, to identify a set of sex-associated genes in this species. We aligned Expressed Sequence Tags (ESTs) from sexual and vegetative tissue of *N. intermedia* to orthologs from three closely related species: *N. crassa*, *N. discreta* and *N. tetrasperma*. The resulting four-species alignments provided a dataset for molecular evolutionary analyses. Our results confirm a general pattern of rapid evolution of fungal sex-associated genes, compared to control genes with constitutive expression or a high relative expression during vegetative growth. Among the rapidly evolving sex-associated genes, we identified candidates that could be of importance for mating or fruiting-body development. Analyses of five of these candidate genes from additional species of heterothallic *Neurospora* revealed that three of them evolve under positive selection.

**Conclusions:**

Taken together, our study represents a novel finding of a genome-wide pattern of rapid evolution of sex-associated genes in the fungal kingdom, and provides a list of candidate genes important for reproductive isolation in *Neurospora*.

## Background

Genes involved in sexual reproduction are found to evolve rapidly in diverse taxonomic groups (e.g., [1-3]). The phenomenon is especially well studied in animals, where rapidly evolving genes are found to be expressed in virtually all stages of reproduction, from mating to fertilization [1]. Large-scale evolutionary analyses show that, in general, genes expressed in animal reproductive organs show a higher divergence than genes expressed in other tissues [4-7]. Examples of rapid evolution are also seen in self/non-self recognition genes in plants [8,9] and in certain reproductive genes of a wide range of eukaryotic taxa, including algae and fungi [3,10-12]. The rapid evolution of reproductive genes is generally assumed to be adaptive [1] and in line with this assumption, positive selection as the driving force has been confirmed for many fast-evolving reproductive genes (e.g., [5,8,13-16]). Multiple hypotheses have been proposed to explain the rapid evolution of reproductive genes in animals and plants, including sexual selection, sexual conflict, sperm competition, self/non-self recognition and selection for reinforced prezygotic mating barriers between species (e.g., [1,3,17]).

Despite the widespread observations of fast-evolving reproductive genes within the animal kingdom, the rate of evolution of genes involved in sexual reproduction of fungi has scarcely been examined. A few examples of fungal reproductive genes that evolve rapidly have been reported, including the mating-type genes, pheromone precursor and receptor genes in filamentous ascomycetes [12,15,18,19]; for certain genes, this rapid evolution has been shown to be driven by positive selection [15,18,19]. Previous genomic comparisons between yeast species have indicated a high turnover of genes involved in sexual reproduction [20,21]. However, there have been no large-scale evolutionary analyses of genes involved in sexual reproduction in other fungi. Thus, it has been impossible to draw conclusions about a general pattern of rapid evolution of reproductive genes in this kingdom.

In this study, we identified and sequenced genes with relatively high expression during early mating between sexually compatible strains of the heterothallic (self-sterile) filamentous ascomycete *Neurospora intermedia*. Genomic data is at present not available for *N. intermedia*, and choosing it for our study made it possible to use our new data together with available genomic data from closely related *Neurospora* species to confirm rapid evolution of sex-associated genes in this fungal genus. In heterothallic species of *Neurospora*, individuals of two different mating types (*mat A* and *mat a*, respectively) must meet in order to enter the sexual cycle. Distinct female and male reproductive structures are formed during mating, and individuals of both mating types can play both female and male roles. In the initial step of the mating process, a specialized female hypha (trichogyne) grows towards the male propagule (conidia or hyphal fragment) of the opposite mating type. This attraction is mediated by mating-type dependent pheromone signaling [22-24]. After cell fusion (plasmogamy) the male nucleus is transported through the trichogyne into the developing immature fruiting body (protoperithecium). Here the male and female nuclei line up and in parallel go through several nuclear divisions, before nuclear fusion (karyogamy) takes place. Karyogamy is shortly followed by meiosis, ascospore and fruiting body development, and the active discharge of the mature ascospores. The initial contact between sexual partners is mainly mating-type dependent and compatible individuals of different species of *Neurospora* are able to mate [25]. However, interspecific matings between *N. crassa* and *N. intermedia* show reduced reproductive success as compared to matings between conspecific individuals, indicating post-mating reproductive isolation between these species. Furthermore, reproductive isolation between species is reinforced in sympatry [25,26], suggesting selection against hybridization. The genetic machinery underlying the early mating procedure (i.e., between plasmogamy and karyogamy), when mate recognition genes are functioning, is as yet largely unknown in *Neurospora*.

We identified genes with relatively high expression during the early phase of sexual reproduction in heterothallic *Neurospora*, and confirmed that the general trend of rapid evolution of sex-associated genes was upheld in this genus. Furthermore, among the sex-associated genes, we identified rapidly evolving candidate genes that might be involved in reproductive incompatibilities between taxa, such as the observed reinforcement between *N. crassa* and *N. intermedia*[25,27]. Finally, we used molecular evolutionary analyses to assess whether positive selection was a driving force for the rapid evolution.

## Results

### Identification of sexual, vegetative and constitutive gene categories in *neurospora*

With the goal of distinguishing categories of genes with constitutive expression from those exhibiting high relative expression during sexual or vegetative development, we performed competitive microarray hybridizations between RNA from sexual and vegetative tissue samples of two strains of *Neurospora intermedia*, using the *N. crassa* microarray (a whole-genome-spotted oligonucleotide microarray containing 9,826 open reading frames [28]). Sexual tissue constituted young fruiting structures formed in crosses of the two strains, and vegetative tissue was composed of young mycelia of the separately grown strains.

In total, 6,126 genes, represented by probes on the *N. crassa* microarray, exhibited well-measured signal and could be analyzed. The data have been deposited both in the Filamentous fungal gene expression database (FFGED) [29] with experiment ID 56 and in the NCBI Gene Expression Omnibus and are accessible through GEO Series accession number GSE37034. Of these 6,126 genes, Bayesian analyses of gene expression levels, using UBAGEL 3.6 [30,31], identified 509 genes that showed a significantly higher expression in the sexual tissue than in the vegetative tissue (*P* < 0.05), 589 genes that showed a relative increase in expression in the vegetative sample (*P* < 0.05), and 5,028 expressed genes that were not found to be differentially expressed in our samples. These three groups define our “sexual”, “vegetative”, and “constitutive” gene categories, respectively. In addition, we constructed a subcategory of the sexual category based on a stringent false discovery rate, so that this category only contained the genes with *Q* < 0.10. This subcategory is referred to as “sexual *Q* < 0.1”, and contained 112 genes.

### EST sequencing of *Neurospora intermedia* and the building of four-way gene alignments

We sequenced ESTs from clones of cDNA libraries constructed from RNA of *N. intermedia* sexual and vegetative tissue (1,920 and 768 clones, respectively). Four-way gene alignments were constructed by adding the assembled EST-sequences of *N. intermedia* to 3-way alignments of the publicly available genome sequences for the two heterothallic *Neurospora* species *N. crassa* and *N. discreta*, and the pseudohomothallic (partially self-sterile) *N. tetrasperma*. Of the 1,392 genes of *N. crassa* mapped to by ESTs of *N. intermedia*, 998 yielded 4-way orthologs including *N. crassa*, *N. discrete*, *N. tetrasperma* and *N. intermedia*. Of those 998, eight 4-way alignments were excluded due to lack of regions where the sequences from all four taxa were overlapping. Thus, in total there were 990 4-way single gene alignments without gaps. The mean alignment length for individual genes was 762 bp, on average including 96% of the nucleotides of the new *N. intermedia* EST-sequence and 62% of the previously aligned sequences of the other three species.

A majority of the genes in the 4-way alignments fell into one of the three categories, defined based on the microarray experiment: of the 990 genes, 99 (10%), 94 (9%) and 628 (63%) fell into the sexual, vegetative and constitutive gene categories, respectively. The remaining 17% (169 genes) did not have an identified category (i.e., data from the microarray analysis is missing for these genes). The subcategory “sexual *Q* < 0.1” contained 26 (2.6%) genes with a 4-way alignment.

### Identification of rapidly evolving genes by maximum likelihood analyses

**Figure 1:**
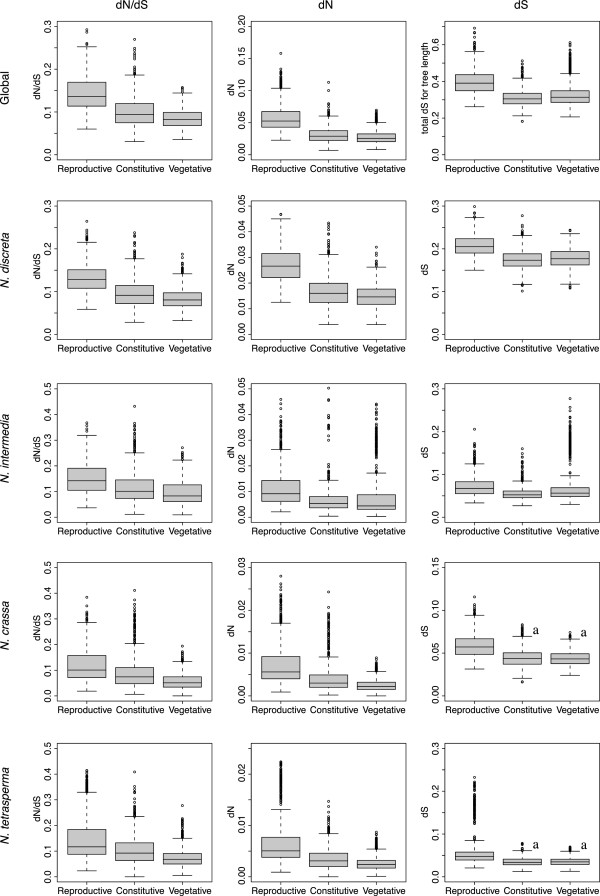
**Boxplots of the distribution of the estimates of dN, dS and dN/dS for the 1000 bootstrap replicates for each gene category.** Data is shown for both the global estimate (one-ratio model in codeml) and for each separate branch (two-ratio model with the specified branch as foreground). Boxes represent the second and third quantile of the estimates and whiskers shows the lowest and highest estimates, excluding outliers. “a” indicates that the two distributions are not significantly different from each other. For all other comparisons *P* < 0.001. Outliers are shown as circles.

To analyze the molecular evolution of genes expressed constitutively or relatively highly during sexual or vegetative growth, we used the maximum likelihood program codeml included in the PAML package version 4.3 [32,33]. The program codeml calculated dN/dS, the ratio of non-synonymous substitutions per non-synonymous site (dN) to synonymous substitutions per synonymous site (dS) over branches in the phylogeny and/or over codons in a gene. We used the 4-way alignment described above to estimate i) the overall dN/dS, dN and dS for the genes in different categories, by using global models of dN/dS among sites and branches, and ii) branch-specific evolutionary rates by using a two-ratio model of dN/dS, in which the branch of interest was specified as foreground and all remaining branches as background. By this approach, we identified 204 genes as more rapidly evolving (exhibiting higher dN/dS) than the mean for the complete phylogenetic tree and/or in any of the branches. Each of the genes and branch(es) for which a dN/dS higher than mean was found, is shown in Additional file 1: Table S1 and summarized in Table 1. Of the 204 rapidly evolving genes, 164 were found to be rapidly evolving using the global model, i.e., the global rate estimated for all four *Neurospora* branches together was significantly higher than the mean (Table 1). These genes were categorized as the “rapidly evolving *Neurospora* genes”. We identified 49 rapidly evolving genes in the branch delineating *N. intermedia*, 41 in the *N. crassa* branch, 135 in the *N. discreta* branch, and 21 in the *N. tetrasperma* branch (Table 1). The number of branch-specific rapidly evolving genes in the phylogeny is lower in the shorter branches (indicated by branch specific dS, Figure 1), which is expected since the power to estimate dN/dS is lower for genes exhibiting very few nucleotide changes. Most of the genes found to be rapidly evolving in each of the separate branches were also found among the rapidly evolving *Neurospora* genes (Additional file 1: Table S1).

**Table 1:** Proportions of individual genes in each category that are found to be rapidly evolving in any of the codeml analyses

**Gene category**	**Number of genes**	**Rapidly evolving genes**					
		**Total**	**Global**	**Branch specific**			
			***Neurospora***	***N. intermedia***	***N. crassa***	***N. discreta***	***N. tetrasperma***
Total	988	204	164	49	41	135	21
Sexual	99	26	19	7	4	22	1
Constitutive	627	112	94	25	22	72	13
Vegetative	94	15	9	2	2	11	1
n/a^b^	168	51	42	15	13	30	6

^b^ genes for which data from the microarray analysis is lacking.

Both total number and the distribution of these genes in the global ratio model and all separate branches is shown.

### Distribution of rapidly evolving genes in gene categories

Of the 164 rapidly evolving *Neurospora* genes, 19 were found in the sexual gene category (Table 1). A higher proportion of genes in the sexual gene category were rapidly evolving (26 of 99: 26%) as compared to the constitutive gene category (112 of 627: 18%) (Table 1, Fisher exact test, two-tailed, p = 0.036). The pattern was similar in the comparison between the sexual and vegetative gene categories (15 of 94: 16%) (Table 1). However, the difference did not pass our criterion for statistical significance (*P* = 0.057). A high proportion of rapidly evolving genes were also found among the genes that were not well-measured on the microarray (51 of 168: 30%) (Table 1), possibly explained by them being too diverged to adequately hybridize to the *N. crassa* oligonucleotide microarray.

### The sex-associated genes evolve faster than vegetative and constitutively expressed genes

To assess whether genes in the sexual gene category evolved faster than the genes of the vegetative and constitutive gene categories, 1000 bootstrap replicates of dN/dS values from a concatenated alignment of 10 randomly chosen genes from each gene category were run. The results are summarized in Table 2 and the distribution of the estimated dN/dS, dN, and dS from the bootstrap analyses are shown as box plots in Figure 1. For both the global analysis and for each branch in the star phylogeny of the 4 species, the mean dN/dS for the sexual gene category was higher than for the constitutive gene category, which in turn exhibited a higher mean estimate of dN/dS than the vegetative gene category (Table 2). A Wilcoxon rank-sum test verified that the distributions of dN/dS estimates for each gene category deviated significantly from each other for both the global model and for each branch separately (*P* < 0.001, Figure 1). The same pattern of sexual > constitutive > vegetative was revealed in examination of the estimated dN, with one exception: in the *N. intermedia* branch, the mean of the vegetative gene category was higher than the mean for the constitutive gene category (Table 2). The median, however, was lowest for the vegetative gene category (Figure 1). The distribution of dN for each gene category (sexual, vegetative and constitutive, respectively) was significantly different from the distributions of dN for both other categories, as calculated for both the global and for each branch separately (Wilcoxon rank sum test, *P* < 0.001).

**Table 2:** Mean dN/dS of 1000 bootstrap replicates of 10 randomly chosen and concatenated genes for each gene category

	**All genes (990)**	**Sexual (99)**	**Constitutive (628)**	**Vegetative (94)**
Global	0.111	0.142	0.099	0.084
*N. intermedia*	0.125	0.151	0.115	0.098
*N. crassa*	0.102	0.120	0.088	0.058
*N. discreta*	0.106	0.131	0.096	0.084
*N. tetrasperma*	0.110	0.152	0.102	0.076

The number of genes in each gene category is shown in parenthesis.

In all analyses (global and per branch) the sexual gene category had a higher mean dS than the other categories, and the difference was statistically significant (*P* < 0.001; Figure 1). For the global model, and the *N. discreta* and the *N. intermedia* branches, the mean dS of the vegetative category was higher than the constitutive category, and the two distributions were statistically significantly different (*P* < 0.001, Wilcoxon rank sum test, Figure 1). In the branches delineating *N. crassa* and *N. tetrasperma*, the vegetative dS distribution did not differ significantly from that of the constitutive category (Figure 1).

In parallel, we performed the same analyses on the smaller subcategory of the sexual category, the “sexual *Q* < 0.1”. All distributions found to be statistically significantly different between the “sexual” category and any of the constitutive and vegetative categories were also found to be statistically significantly different for the “sexual *Q* < 0.1” category (*P* < 0.001) and the differences in mean were always in the same direction (higher for the “sexual *Q* < 0.1” than for the constitutive and vegetative). Thus, the false discovery rate cut off did not change our results.

### Annotation of rapidly evolving sex-associated genes

Table 3 shows the 20 genes that were categorized as sex-associated, and that also had a higher than mean dN/dS in the global analysis (19 genes) and/or in the *N. intermedia* branch analysis (1 unique gene). Of these “rapidly evolving sex-associated genes”, 13 encode proteins that are similar (E-value < 1e-10) to proteins or domains with a characterized function (Table 3). Four hypothetical proteins were predicted to contain secretion signal peptides, transmembrane domains or GPI-anchors and 7 were classified as encoding hypothetical proteins because they displayed low similarity (E-value ≥ 1e-10) to previously characterized proteins or domains (Table 3).

**Table 3:** **Rapidly evolving sex-associated genes in the global model, and/or in the branch delineating *Neurospora intermedia***

**Gene**	**One ratio dN/dS**	**Putative function/Conserved domains**	**E-value**^**a**^
NCU01720^b^	0.583	Glycosyl hydrolase catalytic core protein	2e-49
NCU02916	0.233	Endoglucanase II, glycosyl hydrolase family 61	1e-94
NCU03013^b^	0.386	Anchored cell wall protein-10, Cu,Zn-superoxide dismutase	2e-44
NCU03584^b^	0.176	Polyketide synthase	0
NCU03861	0.245	Glutaminase A	0
NCU04034	0.261	Hypothetical protein, 4 transmembrane domains	-
NCU04628	0.442	C2H2-type zinc finger domain protein	6e-44
NCU04645	0.178	Hypothetical protein, DUF124 domain-containing protein	5e-45
NCU04730	0.168	Post-transcriptional silencing protein QDE-2	0
NCU05191	0.252	Hypothetical protein, signal peptide, 2 transmembrane domains, GPI-anchor	-
NCU05861	0.161	NF-X1 finger and DNA/RNA helicase domain protein	0
NCU06387^b^	0.531	Hypothetical protein	-
NCU07311^b^	0.379	Hypothetical protein, signal peptide, 3 transmembrane domains	-
NCU07743	0.244	Taurine catabolism dioxygenase TauD	1e-81
NCU07888	0.198	Pleckstrin homology domain-containing protein	2e-40
NCU08435	0.379	RNA-dependent RNA polymerase	0
NCU08986	0.257	Hypothetical protein	-
NCU09099	0.267	Hypothetical protein, signal peptide, GPI-anchor	-
NCU09357^c^	0.349	ATPase, DNA/RNA helicase, nonsense-mediated mRNA decay protein	0
NCU09575	0.180	Sterol esterase	0

^a^ E-value originate from BLASTP analysis of NCBI nr database, or from the SMART protein analysis tool. E-values > 1e-10 are not reported.

^b^ Candidate genes selected for further examination for site-specific positive selection by sequencing and analysis of additional heterothallic species of *Neurospora*.

^c^ This gene only had a significantly higher than mean dN/dS for the branch delineating *N. intermedia*.

### Sites evolving under positive selection in candidate sex-associated genes

To provide site-specific estimates of the evolutionary processes underlying the rapid evolution of five of our candidate fast-evolving sex-associated genes, we increased our statistical power by analyzing the genes from additional species of *Neurospora* (Additional file 2: Table S2), again applying the maximum likelihood program codeml included in the PAML package version 4.3 [32,33]. The five candidate genes were chosen from the list of rapidly evolving sex-associated genes (Table 3) based on their higher than mean dN/dS and existence of suitable primer sites in the 4-way alignment. The additional taxon sampling yielded enough power to demonstrate that three of these genes, NCU03013, NCU06387, and NCU07311, contained up to three individual codons that have evolved under positive selection in *Neurospora* (Table 4). In NCU03013, putatively encoding a superoxide dismutase, the two positively selected sites P_192_ and A_196_ are located outside the Cu_Zn_Superoxide_Dismutase family module on the C-terminal side (Additional file 3: Figure S1). Both NCU06387 and NCU07311 are reported as conserved hypothetical proteins and no functional modules were found that can be correlated with the sites under positive selection. However, three transmembrane domains were identified in NCU07311 and two positively selected sites (Y_119_ and A_126_) are located in a predicted extracellular loop and hence putatively exposed to the external environment (Additional file 3: Figure S1). For NCU01720 and NCU03584, we did not detect signs of positive selection at the level of individual codons (Table 4).

**Table 4:** Summary statistics and parameter estimates from analyses of positive selection for selected rapidly evolving sex-associated genes

**Gene name**	**Number of taxa**	**Locus size**^**a**^	**Model**	**lnl**	**p-value**	**Estimated parameters**			**Mean dN/dS**	**Codons**^**b**^
						**dN/dS (ω0)**	**dN/dS (ω1)**	**dN/dS (ω2)**		
NCU01720	12	273	M1a	−1755.83		ω0: 0.0296, p0: 0.7505	ω1: 1, p1: 0.2495		0.272	
			M2a	−1755.47	0.7012	ω0: 0.0368, p0: 0.7603	ω1: 1, p1: 0.2373	ω2: 35.1811, p2: 0.0024	0.349	0
			M7	−1755.82		p: 0.0458	q: 0.1244		0.270	
			M8	−1755.82	1.00	p0: 0.8107	p: 0.0591, q: 0.5334	ω: 1	0.270	0
NCU03013	12	195	M1a	−1298.67		ω0: 0.0130, p0: 0.7967	ω1: 1, p1: 0.2033		0.214	
			M2a	−1290.09	0.0002	ω0: 0.0280, p0: 0.8068	ω1: 1, p1: 0.1769	ω2: 10.1583, p2: 0.0163	0.365	2
			M7	−1298.70		p: 0.0088	q: 0.0284		0.211	
			M8	−1289.99	0.0002	p0: 0.9834	p: 0.0628, q: 0.2608	ω: 9.9750	0.356	3
NCU03584	7	343	M1a	−2157.94		ω0: 0, p0: 0.8807	ω1: 1, p1: 0.1193		0.119	
			M2a	−2157.20	0.4788	ω0: 0, p0: 0.8848	ω1: 1, p1: 0.1014	ω2: 3.1613, p2: 0.0138	0.145	0
			M7	−2158.31		p: 0.0064	q: 0.0427		0.102	
			M8	−2157.38	0.3965	p0: 0.8943	p: 0.005, q: 1.9927	ω: 1.3373	0.141	0
NCU06387	12	407	M1a	−3150.19		ω0: 0.0779, p0: 0.7367	ω1: 1, p1: 0.2633		0.321	
			M2a	−3144.92	0.0051	ω0: 0.1610, p0: 0.8954	ω1: 1, p1: 0	ω2: 2.4839, p2: 0.1046	0.404	0
			M7	−3151.76		p: 0.0984	q: 0.194		0.336	
			M8	−3144.94	0.0011	p0: 0.8963	p: 19.2341, q: 99	ω: 2.4946	0.404	2
NCU07311	11	174	M1a	−1345.32		ω0: 0, p0: 0.6612	ω1: 1, p1: 0.3388		0.339	
			M2a	−1340.47	0.0078	ω0: 0.1134, p0: 0.8530	ω1: 1, p1: 0.0171	ω2: 3.1712, p2: 0.1299	0.526	1
			M7	−1345.56		p: 0.0093	q: 0.0189		0.303	
			M8	−1340.47	0.0061	p0: 0.8661	p: 3.8329, q: 27.2819	ω: 3.1319	0.525	3

^a^Number of analyzed codons

^b^Number of positively selected codons identified with the models

### Phylogenetic specificity and chromosomal locations of genes in different gene categories

Rapid evolution of sex-associated genes would manifest as enrichment of genes in the sexual gene category among those that were found previously to be lineage specific. Correspondingly, vegetative and constitutive genes would be enriched among more phylogenetically conserved gene classes [4,7,34]. Thus, we investigated the proportions of the individual genes falling into different phylogenetic specificity classes (as defined in [35]), and found that they differed between the gene categories in line with these expectations (Table 5). Specifically, the two phylogenetically most specific classes, *Neurospora* orphans and Pezizomycotina-specific genes, were enriched (*P* < 0.01) for sex-associated genes, and the majority of the sex-associated genes (60%) were found in these two classes (Table 5). Furthermore, the phylogenetically broadest gene class (the Eukaryote/Bacterial core) was enriched with genes within the vegetative category (*P* < 0.001, Table 5). A noteworthy exception is that one of the more specific classes, the Pezizomycotina-specific class, was enriched with genes of the constitutive gene category (*P* < 0.001, Table 5). This result is not in accord with the expectation. Furthermore, for the subcategory “sexual *Q* < 0.1”, we did not find a statistically significant enrichment in any of the phylogenetic specificity classes, although the *Neurospora* orphans and Pezizomycotina-specific genes together comprised the majority of the “sexual *Q* < 0.1” genes (51%).

**Table 5:** Phylogenetic specificity of the genes in each gene category

**Phylogenetic class**^**1**^		**Sexual**	**Constitutive**	**Vegetative**
*Neurospora* orphans	Obs	100***	656	54
	Exp	67.3	664.7	77.9
Pezizomycotina-specific	Obs	206**	1771***	117
	Exp	174.1	1718.5	201.4
Ascomycota core	Obs	6	91	9
	Exp	8.8	87.0	10.2
Dikarya core	Obs	68	582	73
	Exp	60.1	593.3	69.5
Eukaryote/Prokaryote core	Obs	77	1448	290***
	Exp	150.9	1489.5	174.6
Other/unknown	Obs	17/35	90/387	17/29
	Exp	10.3/37.5	101.8/370.1	11.9/43.4

^1^Phylogenetic class from Kasuga et al. [35] are defined as the level of specificity in the tree of life at which homologues of the *N. crassa* genes are found in genomes of other organisms.

* *P* <0.05, ** *P* <0.01, *** *P* <0.001: p-values for enriched classes are due to Fisher exact test with Benjamini and Hochberg multiple testing correction.

When investigating the genomic distribution of genes of different categories, we found no statistically significant difference between observed and expected number for any of the gene categories on any of the chromosomes (Fisher’s exact tests, *P* > 0.05, data not shown). Furthermore, we did not find differences in codon usage between the gene categories (data not shown).

### Correlation between gene length, gene expression and evolutionary rate

The distribution of per-gene estimates of mean absolute expression for the constitutive gene category (mean = 1089, median = 365) was significantly different from the distribution for both the sexual (mean = 1703, median = 536) and the vegetative (mean = 1202, median = 546) categories (Wilcoxon rank sum test, *P* < 0.001), while the distribution did not differ significantly between the sexual and vegetative categories (*P* = 0.13). However, we found no statistically significant correlations between the mean absolute expression for each gene and estimated global dN/dS; neither for all genes nor within each category (*R*^2^ ≤ 0.006, *P* > 0.05). The distribution of the length of the gene coding regions (CDS) based on *N. crassa* did not differ significantly between the gene categories (Wilcoxon rank sum test, *P* > 0.05) and we found no significant correlations between CDS length and estimated global dN/dS, for either all genes or within each gene category (*R*^2^ < 0.003, *P* > 0.05). Thus, the observed differences in dN/dS cannot be explained by differences in protein length or absolute expression levels between genes of different categories.

## Discussion

We have demonstrated that the previously observed pattern of high rates of evolution in reproductive genes extends to the fungal kingdom. Genes up-regulated during sexual reproductive stages in *N. intermedia* exhibited a higher mean dN/dS than genes with relatively high expression in vegetative stages or genes that were constitutively expressed. Thus, the examples of rapidly evolving reproductive genes that were previously revealed in fungi [12,15,18,19] can be considered components of a genome level phenomenon, in accordance with the pattern found in the animal kingdom [1,3]. To our knowledge, this study represents the first genome-scale demonstration of the overall rapid molecular evolution of sex-associated genes in a fungal system.

The sex-associated genes identified in this study exhibited higher mean dN/dS than other genes, indicating rapid evolution on the protein level. The concatenated data from 4 species was sufficient to infer the overall evolution of gene categories, but to few taxa were sampled to infer specific codons evolving under positive selection [36]. To further analyze the basis of the higher dN/dS, we sequenced more taxa for five of the rapidly evolving sex-associated genes and demonstrated that three of these genes contain specific codons that have evolved under positive selection. Our results indicate that positive selection is indeed a factor driving the elevated rate of evolution of the sex-associated genes. However, for the majority of the genes in the sexual gene category, we could not distinguish between directional or stochastic processes as the reason for the increased divergence. For most individual genes identified as rapidly evolving, the estimated dN/dS was below 1, meaning they did not show a clear sign of positive selection averaged across sites. Due to the extensive functional constraints of most proteins, in most circumstances only a fraction of all codons in a gene can evolve under positive selection. Thus, genes with a high dN/dS that deviates significantly from the mean are candidates for positive selection even if the dN/dS is below 1 since the probability of positive selection acting on specific codons increases with higher overall dN/dS estimates (e.g., [6]).

Although data from the five candidate genes suggest that positive selection is at least one of the factors leading to an elevated dN/dS in sex-associated genes, a high dN/dS may also be caused by relaxed selective constraints, and we cannot reject this factor as an alternative/additional cause of the elevated dN/dS of the sex-associated genes observed in this study. First, a mutation that is not deleterious in one tissue may be deleterious in another, and hence selective constraints on a gene increase with the number of tissues where it is expressed. Relaxed selective constraints can thus be expected in genes with narrow domains [37], such as those for which expression is specific to the sexual stage in *Neurospora*. Second, one may speculate that *Neurospora* spends a relatively high proportion of its life cycle in vegetative growth [38] and that this leads to greater selective constraints on genes expressed in vegetative mycelia as compared to those induced during sexual reproduction. However, both alternative explanations for our results are contradicted by the finding that genes that are found in the constitutive gene category, i.e., genes with broadest function, do not show the lowest dN/dS.

In addition to a higher dN/dS, genes exhibiting relatively high expression in sexual tissue are characterized by both higher estimated dN and higher estimated dS compared with the constitutive and vegetative gene categories. These rates demonstrate that sex-associated genes are fast evolving at both non-synonymous and synonymous sites, a finding that is consistent with animal systems where high substitution rates in sex-associated genes are reported [2,7]. Variation in mutation rates, which results in variation of dS, is influenced by factors such as GC content, recombination rate, gene conversions and indel frequency [39,40]. Furthermore, a low dS as in the constitutive and vegetative gene categories can be caused by selection for preferred codons (codon bias) to ensure efficient and accurate translation in highly expressed genes [41-43], but we did not find general differences in codon usage between the gene categories. Interestingly, the dS is higher in the genes that are up-regulated in the vegetative tissue in our study compared to the constitutively expressed gene category (although we found no significant difference in the two shortest branches). Further analyses of mutation rate are needed in order to resolve the effect of mutation rate on dS.

We found that the sex-associated genes are enriched in the two phylogenetically most specific gene groups (the *Neurospora* orphans and the Pezizomycotina-specific genes [35]), indicating that they are overrepresented by novel, or ancient and highly divergent, genes. The enrichment of young genes is in line with studies on animals where sex-associated genes are found to show high levels of novelty as well as high birth and death rates [4,7,34] and indicating an over-all rapid evolution of the sex-associated genes on the genome level. Further support of high levels of novelty in fungal sex-associated genes is provided by recent observations based on genomic comparison of yeasts: many genes essential for sexual reproduction in *Saccharomyces cerevisiae* seem to be absent in sexually reproducing *Candida* species [20,21,44]. Although the genes investigated in yeasts function in determining cell identity and during meiosis, thus presumably operating at different developmental stages than the genes investigated herein, our studies point in the direction of a high turnover of fungal genes involved in sexual reproductive processes. We note that 57% of the rapidly evolving sex-associated hypothetical proteins are predicted to be surface exposed due to the presence of secretion signal peptides, transmembrane domains, or GPI-anchors. This finding indicates that these proteins interact physically with extracellular proteins or substrates.

The wide array of rapidly evolving reproductive genes in animals and plants is generally explained by a set of different theories that include sexual selection (in the form of sperm competition, co-evolution of male and female genes caused by sexual conflict, female choice), self/non-self recognition to avoid inbreeding [1] and/or reinforcement of mating barriers between species [17]. In contrast to animals, reports on sexual selection in fungi are exceptional [45-47]. As a consequence, there is a general lack of knowledge of the effects of sexual selection in fungal biology, although recent evidence of both female choice and male-male competition has been shown in the basidiomycete *Schizophyllum commune*[45]. There is no inherent reason why sexual selection should not be applicable to the fungal kingdom, and we suggest that it plays a role in driving the observed divergence of the sex-associated genes in *Neurospora*. Indeed, the scarcity of studies demonstrating sexual selection in fungi may be attributable to the difficulty of observing the traits that are under selection: relevant traits are likely to be apparent only at cellular or subcellular levels.

While the male partner in *Neurospora* contributes solely with the fertilizing nucleus, the maternal parent is alone responsible for the allocation of resources to develop the fruiting body and ascospores. If the female is likely to make contact with more than one potential partner, the evolutionary cost of a bad choice can be severe, especially since a mycelium is inhibited from mating again after the mating process is completed [26,48]. Extensive crossing experiments performed with the aim of distinguishing between biological species of *Neurospora*[25,49] demonstrate notable differences in mating success within species. One experiment has shown evidence of reinforced reproductive barriers between sympatric populations of *N. crassa* and *N. intermedia*[25,26], in the form of earlier abortion of sexual development in hybrid crossings in sympatric populations compared to allopatric. The mechanism by which the hybrid crosses are aborted is unknown, but it seems to operate after mating and before karyogamy [25,26], which is the time span from which our sex-associated gene category was identified. Thus, the sex-associated genes identified in this study may be affected by female choice. In *Neurospora*, there is neither sperm fluid nor specific male organs, and fertilization is possible by nuclear transfer from hyphal fragments acting as male fertilizing units. In addition, the expressed genes identified in the current study most likely originate from the female partner, due to heavy overrepresentation of protoperithecial tissue compared to the low amounts of cells in the conidial suspension used for fertilization. Nevertheless, until male and female tissues are analyzed separately, it is not possible to disentangle whether the higher rate of divergence observed in sex-associated genes in this study can be explained by male-specific rapid evolution.

The primary goal of this study was to test whether evolutionary rate differed in vegetative and sex-associated genes. Our result that sex-associated genes have evolved faster is robust to differing stringency of categorization based on the expression-data. Future studies may reveal the exact driving force and the functional consequences of the changes. Several recent studies indicate that initiation and development of the fungal fruiting body is a complex process that involves a specific regulatory program (e.g., [50-53]). Nevertheless, investigations of the processes underlying rapid evolution of sex-associated genes in fungi are severely hampered by the scarcity of functional studies of fungal mating. With the exception of mating-type genes [54], pheromones and their receptors [23,24,55], limited information is available about the proteins and genes involved in the mating process in *Neurospora*. Accordingly, functional data are lacking for the vast majority of the sex-associated genes investigated in this study, and they are solely identified based on the assessments of gene expression. This lack of annotation makes it probable that our recognized set of sex-associated genes contain some genes that are not important for mating. Additional experimentation is needed to confirm their importance in the sexual reproductive process in *Neurospora*. Moreover, our discretely staged experimental design makes it possible that some transiently expressed genes are missed. Nevertheless, in the literature we find phenotype data relating to sexual development for mutants of two of the rapidly evolving sex-associated genes, NCU04628 and NCU03584. NCU04628 is predicted to encode a C2H2-type zinc finger protein, disruption of which results in abnormal development of perithecia and ascospores [56]. NCU035684 encodes a putative homolog to the *Sordaria macrospora pks* polyketide synthase gene. Disruption of this homolog in *S. macrospora* results in an albino phenotype and fragile, but viable, ascospores [57].

Microarray data was not well-measured for 169 of the genes with a 4-way alignment. The reason for the missing data may be sequence differences between *N. intermedia* and *N. crassa* that result in absence of signal during hybridization, which would result in absence of gene category data for the most rapidly evolving genes. This explanation is supported by the fact that we found the highest proportion of individual genes with a higher than mean dN/dS in this group of genes. In addition, our criterion for construction of the 3-way and four-way alignments may have excluded the most divergent genes from our analyses. We cannot deduce whether the inclusion of these “invisible” orthologs in the analyses would have changed any of our results.

## Conclusion

Taken together, this study extends the domain of the genome-wide pattern of rapidly evolving reproductive genes from animal systems to fungi. Our results demonstrate that positive selection is at least one of the factors driving this rapid evolution. Although the precise cause for positive selection of sex-associated genes in *Neurospora* is unknown, we speculate that it may be driven by natural or sexual selection. Sex-associated genes that are rapidly evolving between taxa are interesting as candidates for future studies as they may play a role in reproductive incompatibility between *Neurospora* species. Correspondingly, the finding that sympatric interspecies matings in *Neurospora* abort earlier than allopatric interspecies matings [25,26] suggests that mate recognition genes are under selection; a pattern consistent with reinforcement of reproductive barriers [25,26]. Future studies addressing the functional roles of the identified genes during reproduction are needed in order to clarify the mechanisms that ultimately result in higher rate of change for sex-associated genes. This functional knowledge will enable us to investigate the generality of diverse theories proposed to explain the rapid evolution of sex-associated genes across kingdoms.

## Methods

### Strains of *Neurospora intermedia* used in the study

Two strains of *Neurospora intermedia*, FGSC 8782 and FGSC 8882, were used in this study. FGSC 8782 is of mating-type *a* and was originally collected in Homestead, Florida, U.S.A., while FGSC 8882 is of mating-type *A*, and was collected in Puerto Cortes, Honduras. Both strains belong to the phylogenetic subgroup NiA of *N. intermedia*[58]. The two strains are highly inter-fertile and originate from a population that shows reinforced reproductive isolation with sympatric strains of *N. crassa*[25]. In addition, we used representatives of twelve heterothallic (self-sterile) species and subspecies (Additional file 2: Table S2). All strains were obtained from the Fungal Genetics Stock Center (FGSC, University of Missouri, MO, USA).

### Preparation of vegetative and sexual tissue samples of *Neurospora intermedia*

Cultures of *N. intermedia* for microarray analysis and EST sequencing were grown in 90-mm Petri dishes on solid synthetic crossing medium (SCM) [59] with 2% sucrose, covered by a layer of sterilized cellophane membrane, and in test tubes with Vogel’s minimal medium [60] with 1.5% sucrose. Unless otherwise specified, cultures were grown at 25°C.

The sexual sample was obtained by collecting tissue from reciprocal crosses between the two isolates. The strain to be the perithecial (maternal) parent of each cross was incubated on SCM until the Petri dish contained numerous protoperithecia (the unfertilized female reproductive structures; 11 days for FGSC 8782 and 13 days for FGSC 8882). For the production of conidia (the male fertilizing unit), each of the strains was grown on Vogel’s media for 3 days at 37°C. The conidia were collected with a spatula and suspended in water. The conidial concentration of the suspension was estimated by a hemacytometer and adjusted to 500,000 conidia per mL. During fertilization, 0.5 mL of the suspension (about 250,000 conidia) was spread with a sterile glass spreader onto the Petri dish containing the maternal tissue of the opposite mating-type. Fertilization was performed by transferring conidia from the *mat a* strain (8782) to the Petri dish containing maternal tissue of the *mat A* strain (8882), and *vice versa*, to produce two reciprocal crosses. After fertilization, the crosses were incubated in darkness until harvest. The sexual tissue was sampled at five different time points: 3, 12, 24, 36, and 48 hours after the conidial suspension was added. This time series had previously been verified by microscopy to represent tissue at the stage of development until karyogamy (i.e., presence of croziers [61]) under our growth conditions. At harvest, the tissue was scraped off the cellophane with a scalpel and collected in 1.5 ml Eppendorf tubes. The developing perithecia of the harvested tissue were not separated from the surrounding hyphae, and thus, the sexual tissue contained a background level of vegetative tissue. Each tube was immediately snap frozen after harvest in liquid nitrogen and stored at −70°C. Samples (RNA or tissue, see below) from the 5 time points from the two strains were pooled in equal amount and constitute our sexual sample in subsequent microarray and EST-analyses.

The vegetative tissue of each strain was grown for three days on SCM in constant darkness. At the time of harvest, the mycelia were inspected under a dissecting microscope to verify absence of protoperithecia and conidia in the culture. Thus, the same media and culture conditions were used for sexual and vegetative tissues, and the developmental stage of the tissue was the only difference between them. The same method for harvesting was used as with the sexual tissue. A pooled sample of RNA or mycelia of the two strains was used as the vegetative sample in subsequent microarray and EST-analyses, respectively (see below).

### RNA and cDNA processing

We followed Clark et al. [62] for RNA and cDNA processing for the microarray study. Briefly, we extracted total RNA from 50–100 mg portions of the tissue using the TRI REAGENT kit (Molecular Research Center, Inc. Cincinnati, OH). The tissue was homogenized by grinding in a 7 mL Dounce glass tissue grinder and by using Qiagen Qiashredder columns (Qiagen, Chatsworth, CA). After extraction, equal amounts (μg) of the total RNA from the sexual tissue of all five time-points from both strains were pooled together to constitute the sexual sample. For the vegetative sample, total RNA from mycelia of each strain was pooled in equal amounts. MRC oligo(dT)-Cellulose columns (Molecular Research Center, Inc. Cincinnati, OH) were used for poly(A) + mRNA purification. Samples for each hybridization were independently subjected to reverse transcription using Superscript II reverse transcriptase (Invitrogen), 0.5 μg oligo(dT) primers (Invitrogen), and 2 μg poly-A mRNA.

### Microarray hybridization

The cDNA was coupled with Cy3 or Cy5 labeled probes (Amersham Biosciences, Uppsala, Sweden), then purified using the QIAquick PCR purification kit (Qiagen). Four competitive hybridizations, including two dye-swaps, were performed between cDNA of the sexual and vegetative samples. Hybridizations were made in the dark at 55°C for 16 hours. The microarrays used in this study [28,63] were based on the genome sequence of *Neurospora crassa*, a close relative to *N. intermedia*. The whole-genome-spotted oligonucleotide microarray contained 9,826 open reading frames, each represented by a 70mer oligonucleotide probe that was robotically printed on CMT-GAPS-aminopolysilane-coated glass slides (Corning, Corning, NY) at the Yale University Center for Genomics and Proteomics, following the procedure by Kasuga et al. [28]. The divergence between *N. crassa* and *N. intermedia* is very low, with the proportion of variable sites in coding regions of housekeeping genes being 1.2% [64]. Therefore, we considered this 70-mer array, designed from the *N. crassa* genome sequence, to be appropriate for the analyses of expression of *N. intermedia* isolates, especially when analyzing differences in relative expression between strains of *N. intermedia* and not between *N. intermedia* and *N. crassa*[65,66].

### Microarray data acquisition and analysis

An Axon GenePix 4000B microarray scanner was used to acquire a two-channel color image of the array fluorescence. The microarray spots were located by using the GenePix Pro 6.0 software package (Axon Instruments, Foster City, CA) together with the array list file [67]. Before data collection each microarray was manually screened and adjusted, and abnormal spots were excluded. We normalized the ratio results as in Townsend [68] using the online tool available at The Filamentous Fungal gene Expression Database [29,69]. A Bayesian Analyses of Gene Expression Levels (BAGEL) was performed using the software UBAGEL 3.6 [30,31] on the normalized data. For each gene, the relative expression level and a credibility interval of 95% was calculated.

We used the results from the BAGEL analysis to categorize genes among three exclusive alternatives: sexual, vegetative and constitutive. The sexual gene category contains all genes that exhibited a statistically significantly (*P* < 0.05) higher expression in the sexual tissue relative to the vegetative. The vegetative category contains all genes that exhibited a statistically significant higher expression in the vegetative tissue compared to the sexual, and the constitutive category consisted of all genes that did not exhibit differential expression between the sexual and vegetative samples. *Q*-values were calculated from our *P*-values using the software QVALUE [70] with the default settings. *P* < 0.05 corresponded to *Q* < 0.62 for the sexual category genes, and corresponded to *Q* < 0.51 for the vegetative category genes. Because the aim was to divide the genes into their most likely gene categories, the false discovery rate is not critical (inclusion of inappropriately classified genes would decrease effect size, and therefore be conservative). Nevertheless, we performed an additional analysis on a more strictly defined sexual gene category only including genes with *Q* < 0.10. The *Q*-value cutoff of *Q* < 0.10 was selected to minimize the false positives while still allowing enough genes in the category to convey statistical power in the analyses. We refer to this subdivision of the sexual category as the “sexual *Q* < 0.1”.

### cDNA-library construction and EST sequencing of *Neurospora intermedia*

RNA extraction, subtractive cDNA library construction and EST sequencing were outsourced to Agencourt Bioscience Corporation (Beverly, MA). Total RNA was extracted from both the vegetative and the sexual tissue (pooled in equal amounts from both strains at different time points, see above), by using the Agencourt RNAdvance Tissue Kit. A subtracted cDNA library, enriched for sequences of genes expressed in the sexual sample, was prepared by the Suppression Subtractive Hybridization method [71] in which the cDNA from the vegetative sample was used to subtract the genes shared by the two samples. In addition, a separate library was constructed from the vegetative sample. Sequence data was composed of 5,376 reads, sequenced by using ABI (Applied Biosystems) sequencing technology: 3,840 of which were from the subtracted sexual cDNA library (1,920 clones, sequenced in two directions) and 1,536 of which were sequenced from the vegetative library (768 clones, sequenced in two directions). Thus, 1,920 clones from the subtracted sexual cDNA library and 768 clones from the vegetative library were sequenced in both forward and reverse directions. The EST-data have been deposited in EMBL/NCBI’s EST database, and are accessible through the accession number HE957083-HE961812.

### EST data assembly

The ESTs were processed and assembled *de novo* with the phred/crossmatch/phrap toolchain [72,73]. Basecalling, quality filtering and trimming was carried out with phred v0.071220.b using the default quality cutoff settings, after which crossmatch v1.090518 was used to screen out remaining vectors using the UniVec database [74]. Phrap was used to assemble the sequence with overlapping regions into contiguous sequences referred to here as Phrap contigs. After filtering and assembly, the data consisted of 4,984 reads.

### Three-way alignments of publicly available *Neurospora* sequences

The 3-way alignments were generated from *N. crassa*[75], *N. discreta*[76] and *N. tetrasperma* (version 1 [77,78]) by using the SYNERGY algorithm [79], which identifies orthologs based on the sequence similarity from pair-wise BLAST of each proteome (expectation cutoff < 1E-5) and synteny of loci across the 3 genomes. In total, a 3-way alignment was completed for 5,635 individual genes that were found to be unambiguously orthologous among the 3 species (Stajich et al., unpublished), corresponding to roughly half of the genes in the *N. crassa* genome.

### Orthology search for the *Neurospora intermedia* sequences

A BLAST database [80] was built from the 3-way alignments and the *N. crassa* genome (downloaded on April 13, 2011) and served as reference for establishing orthology for the new and pre-processed *N. intermedia* sequence reads. Gene IDs were assigned to the *N. intermedia* reads by running megaBLAST [81] queries against the 3-way database. The queries were configured to use a length cutoff of 100 aligned bases to register a match. Of the 4,984 reads retained after assembly, megaBLAST against the 3-way database identified 2,573 reads as usable, corresponding to only a single gene target. By running BLAST on the Phrap-generated contigs built from the total read pool, 104 additional reads were identified, and finally, 188 reads were unambiguously identified using the less stringent BLASTN algorithm and an alignment cutoff at 60 bases. The 1,939 remaining sequence reads were not used in the subsequent analyses, but were run in BLAST against the *N. crassa* genome to provide additional gene statistics using the same algorithms, prioritizing megaBLAST hits over BLASTN hits. In total, 4,585 of the *N. intermedia* reads yielded a BLAST-hit to any of 1,392 *N. crassa* genes.

### Adjustments of the 4-way alignments

A custom pipeline was written in Bash/Perl to assemble the identified *N. intermedia* data and align it to the three-way alignments on a gene-by-gene basis. For each of the genes in the 3-way set, we used BLAST to find the best Phrap contig and unassembled singlets to establish direction and alignable boundaries. These cues were used to identify local subalignments against which each Phrap product could be aligned using the G-INS-i algorithm and seed method in MAFFT [82,83]. Frame-shifting inserts were filtered out and incomplete codons or premature stop codons were masked as missing data. The aligned *N. intermedia* data was merged into a single consensus sequence. In cases where aligned fragments were overlapping but had not been assembled into a contig by Phrap, remaining sequence length and contig size were used as tokens of sequence quality to resolve which fragment the consensus should be based on. Finally, the new four-way alignment was filtered so that only gap-less codon positions with data from all four species were included in downstream analyses. The program Gblocks [84] was used to remove poorly aligned regions before further analyses.

### Identification of rapidly evolving genes

To identify rapidly evolving genes in *Neurospora*, we invoked two global ratio models using the maximum likelihood program codeml, included in the PAML package version 4.3 [32,33]: one in which the global dN/dS was estimated as a free parameter, and one in which it was fixed to the mean dN/dS for all genes. The mean dN/dS was estimated by running the codeml analysis on a concatenated alignment of all 4-way orthologous gene alignments. These two models are nested, and differ in one estimated parameter, so a log-likelihood ratio test (LRT) with one degree of freedom was used to to evaluate the fit to the data for the two models, and thereby identify genes with a dN/dS significantly deviating from the mean. In the phylogeny of the four taxa included in the analyses, *N. discreta* represents the most ancestral branch, but the phylogenetic relationship is not fully resolved for the other three taxa [58]. Thus, we used a completely unresolved star tree as an unrooted input phylogeny for the analyses.

To test for branch-specific rapidly evolving genes, i.e., genes evolving rapidly in the branches delineating *N. intermedia*, *N. crassa*, *N. discreta* or *N. tetrasperma*, we used the same approach but with the two-ratio model specifying the branch of interest as foreground and comparing the log-likelihood value with the value for the model where the foreground branch dN/dS was fixed at the mean for that branch. To adjust for multiple testing, *Q*-values were calculated from our *P*-values using the software QVALUE [70] and *Q* < 0.05 was considered significant.

### Estimating global and branch-specific dN/dS for gene categories

To estimate the mean dN, dS, and dN/dS for each gene category distinguished by the microarray experiment (constitutive, sexual and vegetative) we implemented a bootstrap approach. For each bootstrap replicate, 10 randomly chosen genes were concatenated. The concatenation procedure was performed to overcome the uncertainty of the estimates typical for short sequences. On each concatenated alignment, we ran both the global ratio model and the four versions of the two-ratio models: one for each branch specified as the foreground branch. For each gene category, 1000 bootstrap replicates were performed, and the mean for the obtained estimates was calculated for each category. To test for differences in the distribution of the estimates for dN, dS and dN/dS between the different categories, a Wilcoxon rank sum test with continuity correction, as implemented in R, was performed, and *P*-values were adjusted for multiple testing using the method described by Benjamini and Hochberg [85]. In addition, these analyses were performed on the small subset consisting of 26 sexual genes falling into the category “sexual *Q* < 0.1”.

### PCR amplification and sequencing of candidate genes for positive selection

Strains for PCR and sequencing (Additional file 2: Table S2) were grown in test tubes with Vogel’s minimal medium with 1.5% sucrose [60] and DNA was extracted as in Karlsson *et al.*[15]. Primers for selected genes were designed based on homologous regions of the 4-way alignment, and are given in Additional file 4: Table S3. PCRs were performed using the Expand High Fidelity PCR System (Roche Applied Science, Indianapolis, USA). PCR products were purified with ExoSAP-IT reagent (Amersham Biosciences, Uppsala, Sweden). Sequencing was performed using the BigDye Terminator v3.1 Cycle Sequencing Kit (Applied Biosystems, Foster City, USA). The products were cleaned using BigDye XTerminator Purification Kit (Applied Biosystems), and then sequenced on an ABI3730XL (Applied Biosystems). The raw sequences were edited using the software package Sequencher 4.1.4 (Gene Codes Corporation, Ann Arbor, USA). The sequence alignments for each gene were adjusted manually using BioEdit version 7.0.0 [86].

### Analyses of positive selection of candidate genes

To test if the higher than mean dN/dS estimates for individual sex-associated genes might be caused by positive selection on individual sites within genes, we chose five genes for additional analysis among a larger set of species, ranging from seven to twelve taxa for the different genes (Additional file 2: Table S2). The genes were selected from the list of rapidly evolving sex-associated genes based on their high dN/dS and the existence of suitable primer sites in the 4-way alignment. To evaluate the selective constraints acting on individual codon sites, we ran four models in the PAML package version 4.3 [32,33]: M1a, M2a, M7 and M8. The models constitute two nested pairs (M1a + M2a and M7 + M8) with one model of each pair only containing site classes allowing dN/dS to vary between 0 and 1 (neutral models; M1a and M7), while the second model of each pair (selection models; M2a and M8) contains an additional site class in which dN/dS ≥ 1, thus allowing positive selection. We based the analysis on the phylogeny of the heterothallic *Neurospora* from Dettman et al. [58]. Before the analysis, we excluded intronic sequences from the alignments and regions for which sequence data for less than half of the taxa were available. The Bayes empirical Bayes (BEB) calculation of posterior probabilities for site classes implemented in model M2a and M8 was used to identify codons likely to have evolved under positive selection [87].

### Functional annotation of rapidly evolving sex-associated genes

Sex-associated genes identified to be rapidly evolving in the global model or in the *N. intermedia*-branch were individually studied. Translated amino acid sequences were analyzed with BLAST at NCBI and for conserved domains using the SMART protein analysis tool [88]. SignalP 3.0 [89] was used to search for signal peptide cleavage sites, and the big-PI Fungal Predictor program [90] was used to search for GPI-anchor sequences.

### Statistical analyses of gene category characters

We investigated the phylogenetic distribution and chromosomal location of the genes of each gene category. The phylogenetic distribution for individual genes was taken from Kasuga et al. [35], which divided annotated protein coding genes from *N. crassa* into phylogenetic specificity classes depending on the phylogenetic relatedness in the tree of life of organisms with homologues of the gene. The chromosomal location was based on the annotation of the *N. crassa* genome ([75] downloaded on March 15, 2011). Over- and under-representation, for both phylogenetic distribution and chromosomal location, across the gene categories, were assessed using Fisher’s exact test. *P*-values were adjusted for multiple testing using the method described by Benjamini and Hochberg [85] as implemented in R. We used the significance level of *P* < 0.05.

Differences in codon usage between the gene categories was analyzed by performing multivariate (correspondence) analysis using the program CodonW version 1.4.4 (http://codonw.sourceforge.net/). The analysis was performed on the *N. crassa* gene sequences from the four taxon alignments used in the dN/dS analysis. Codon usage for gene categories was visualized by plotting the position of each gene on the resulting correspondence analysis axis 1 and 2.

### Correlation between gene expression, gene length and evolutionary rate

Absolute expression for each gene was estimated by calculating the mean of the background-subtracted foreground intensity of the well-measured spots on the microarray [68]. For genes within the sexual and the vegetative gene categories the absolute expression means were calculated on the expression in the specific tissue types only. The mean for each gene in the constitutive category and the estimate for all genes were estimated from all measurements of expression for each gene. To test for differences in the distribution of the per-gene expression between the different categories, a Wilcoxon rank sum test with continuity correction, as implemented in R, was performed. Linear regression was used to calculate the per-gene association between absolute expression and estimated global dN/dS, and between CDS length of *N. crassa* and estimated global dN/dS for each gene category and for the complete dataset. The linear regression analyses were performed in R.

## Competing interests

The authors declare that they have no competing interest

## Authors’ contributions

KN participated in the design of the study, performed the majority of the laboratory work, performed the evolutionary analyses and drafted the manuscript. AW participated in the data assembly and bioinformatics work. NS participated in the laboratory work and data analyses. JS participated in the sequence alignment. JPT contributed reagents, analytical advice, and assisted in drafting the manuscript. MK participated in the design of the study, data analyses and drafting of the manuscript. HJ conceived of the study, participated in study design, coordination, and in drafting the manuscript. All authors read and approved the final manuscript.

## Supplementary Material

Additional file 1: Table S1 A list of rapidly evolving genes, and the branch(es) in which a dN/dS higher than mean are found. Click here for file

Additional file 2: Table S2 Candidate genes used to test for positive selection, and ID of additional heterothallic *Neurospora* strains sequenced. (PDF 51 kb) Click here for file

Additional file 3: Figure S1 Nucleotide and amino acid alignments of the three genes NCU03013, NCU06387, and NCU07311, showing which codons were found to evolve under positive selection. (PDF 13386 kb) Click here for file

Additional file 4: Table S3 Primers used for PCR amplification of candidate genes for test for positive selection. (PDF 34 kb) Click here for file
