# Supplementary material for: Analyses of expressed sequence tags in Neurospora reveal rapid evolution of genes associated with the early stages of sexual reproduction in fungi
Source: BMC Evol Biol. 2012 Nov 27;12:229. doi: 10.1186/1471-2148-12-229 (PMC3571971; doi:10.1186/1471-2148-12-229)
Supplement: Additional file 1: Table S1 — A list of rapidly evolving genes, and the branch(es) in which a dN/dS higher than mean are found. [file 1471-2148-12-229-S1.pdf]

	dN/dS above average						*N. crassa* annotation information				
Gene	global	*dis*	*int*	*cra*	*tet*	Gene category	Length	LG	Start	Stop	Gene Name
NCU00042	x	x				constitutive	1285	LG III	2381584	2382868	selenoprotein domain-containing protein
NCU00052	x					constitutive	2290	LG III	2418803	2421092	alpha-methylacyl-CoA racemase
NCU00127	x	x		x		constitutive	2706	LG III	2716885	2719590	hypothetical protein
NCU00138	x	x	x	x	x	constitutive	3378	LG III	2752660	2756037	
NCU00167	x	x				constitutive	2731	LG III	2867149	2869879	hypothetical protein
NCU00244	x					constitutive	1579	LG III	3154187	3155765	glycosyl transferase
NCU00401	x	x	x			vegetative	2224	LG III	3729613	3731836	hypothetical protein
NCU00434		x				constitutive	2791	LG III	3847356	3850146	protein phosphatase 2C isoform beta
NCU00458	x	x		x		n/a	1364	LG III	3927960	3929323	hypothetical protein
NCU00486	x	x	x			constitutive	3981	LG III	4023237	4027217	hypothetical protein
NCU00517	x	x				n/a	2893	LG I	8147689	8150581	cell cycle checkpoint protein RAD17
NCU00634	x					constitutive	1371	LG I	7746371	7747741	ribosomal protein L14
NCU00716		x				reproductive	1147	LG I	7452365	7453511	non-anchored cell wall protein-5
NCU00761					x	constitutive	1754	LG I	7274308	7276061	triacylglycerol lipase
NCU00774	x	x	x		x	constitutive	1635	LG I	7217862	7219496	hypothetical protein
NCU00821			x			constitutive	2285	LG I	7056000	7058284	sugar transporter
NCU00923	x	x				constitutive	2090	LG I	6710795	6712884	hypothetical protein
NCU00926	x	x	x	x		n/a	2612	LG I	6703376	6705987	hypothetical protein
NCU00938	x	x				constitutive	2064	LG I	6663511	6665574	hypothetical protein
NCU00984	x	x				n/a	3634	LG I	6493485	6497118	von Willebrand domain-containing protein
NCU00985	x					constitutive	2289	LG I	6488910	6491198	hydrolase
NCU00992	x	x	x	x		constitutive	1720	LG I	6466804	6468523	hypothetical protein
NCU01106	x	x	x			constitutive	2162	LG V	2936025	2938186	L-amino acid oxidase
NCU01123	x	x				vegetative	1593	LG V	2972222	2973814	hypothetical protein
NCU01195	x		x			constitutive	1907	LG V	3200932	3202838	amination-deficient
NCU01199				x		constitutive	1303	LG V	3218922	3220224	SNARE domain-containing protein
NCU01202	x					constitutive	1506	LG V	3223676	3225181	hypothetical protein
NCU01270	x					n/a	1716	LG V	3468143	3469858	ribose-phosphate pyrophosphokinase II
NCU01294	x	x				n/a	3628	LG V	3553367	3556994	hydrolase
NCU01309		x				vegetative	2123	LG V	3598720	3600842	hypothetical protein
NCU01418	x					constitutive	1617	LG V	4007703	4009319	clock-controlled gene-6
NCU01449	x		x			n/a	3089	LG V	4705718	4708806	GTP cyclohydrolase II

NCU01559	x			x		constitutive	1400	LG II	3086303	3087702	hypothetical protein
NCU01668	x				x	n/a	2417	LG II	2755579	2757995	hypothetical protein
NCU01670	x		x			constitutive	1483	LG II	2750781	2752263	type 1 phosphatase regulator ypi-1
NCU01720	x	x	x			reproductive	2296	LG II	2644627	2646922	hypothetical protein
NCU01781	x	x		x		constitutive	2463	LG II	2462714	2465176	hypothetical protein
NCU01893	x	x				constitutive	2305	LG I	2121928	2124232	hypothetical protein
NCU01932				x		constitutive	3938	LG I	1970961	1974898	hypothetical protein
NCU01935	x			x		n/a	2226	LG I	1959652	1961877	palmitoyltransferase PFA5
NCU02024	x		x			constitutive	2062	LG I	1640137	1642198	hypothetical protein
NCU02114	x	x				constitutive	3544	LG I	1232089	1235632	G1/S-specific cyclin Cln1
NCU02125	x	x				constitutive	1159	LG I	1164048	1165206	hypothetical protein
NCU02172	x	x				constitutive	4409	LG I	1002515	1006923	hypothetical protein
NCU02187	x	x			x	constitutive	1943	LG I	928672	930614	hypothetical protein
NCU02461	x	x		x		constitutive	1406	LG VII	2148634	2150039	hypothetical protein
NCU02526		x				constitutive	2059	LG I	2626204	2628262	hypothetical protein
NCU02541	x		x			constitutive	2307	LG I	2671491	2673797	oligosaccharyltransferase alpha subunit
NCU02732	x	x	x	x		constitutive	2468	LG I	3358266	3360733	hypothetical protein
NCU02745	x			x		constitutive	1918	LG I	3401488	3403405	hypothetical protein
NCU02853				x		vegetative	3361	LG I	8911982	8915342	C2H2 type zinc finger domain-containing protein
NCU02886	x		x			constitutive	1839	LG I	8803633	8805471	hypothetical protein
NCU02916	x	x				reproductive	1148	LG I	8701896	8703043	endoglucanase II
NCU02979			x			n/a	3527	LG I	8516237	8519763	AMP deaminase
NCU03013	x	x				reproductive	1346	LG I	8441217	8442562	anchored cell wall protein-10
NCU03105	x	x		x		constitutive	1710	LG I	4579422	4581131	hypothetical protein
NCU03153		x				n/a	1826	LG I	4740988	4742813	hypothetical protein
NCU03169			x			n/a	2919	LG I	4777060	4779978	hypothetical protein
NCU03179	x	x			x	n/a	813	LG I	4817014	4817826	hypothetical protein
NCU03191	x	x	x			n/a	1769	LG I	4859024	4860792	rab protein geranylgeranyltransferase component A
NCU03247		x				reproductive	2275	LG I	5049047	5051321	hypothetical protein
NCU03284	x		x			n/a	1798	LG I	5173916	5175713	hypothetical protein
NCU03285	x	x			x	n/a	2258	LG I	5176196	5178453	hypothetical protein
NCU03336	x	x				n/a	3693	LG II	1053305	1056997	hypothetical protein
NCU03584	x		x			reproductive	7496	LG V	1714456	1721951	polyketide synthase
NCU03681	x					constitutive	3154	LG V	2049418	2052571	hypothetical protein

	dN/dS above average						*N. crassa* annotation information				
Gene	global	*dis*	*int*	*cra*	*tet*	Gene category	Length	LG	Start	Stop	Gene Name
NCU03694	x	x				n/a	2199	LG V	2100199	2102397	hypothetical protein
NCU03698	x	x	x	x		n/a	1843	LG V	2115470	2117312	hypothetical protein
NCU03714	x		x	x		n/a	2187	LG V	2180457	2182643	thioredoxin
NCU03741		x				constitutive	2519	LG V	2262588	2265106	NAD kinase/ATP NAD kinase
NCU03755	x				x	constitutive	1718	LG V	2313286	2315003	flavin dependent monooxygenase
NCU03793	x			x		constitutive	1991	LG V	2434991	2436981	hypothetical protein
NCU03810	x	x		x		n/a	985	LG V	2481632	2482616	hypothetical protein
NCU03851	x					constitutive	1437	LG VI	2747393	2748829	hypothetical protein
NCU03861	x	x		x		reproductive	2955	LG VI	2714126	2717080	glutaminase A
NCU03868	x	x				vegetative	2390	LG VI	2672208	2674597	hypothetical protein
NCU03881		x				vegetative	1494	LG VI	2612139	2613632	hypothetical protein
NCU03946	x		x			constitutive	2003	LG VI	2429758	2431760	polyadenylate-binding protein 2
NCU04032	x	x		x		constitutive	1121	LG VI	2124626	2125746	hypothetical protein
NCU04034	x	x				reproductive	1170	LG VI	2119686	2120855	hypothetical protein
NCU04063	x		x			constitutive	1555	LG VI	2010361	2011915	protein transporter sec-13
NCU04074	x		x			constitutive	1715	LG VI	1977426	1979140	NADH:ubiquinone oxidoreductase 30.4
NCU04101		x				constitutive	3192	LG V	4827488	4830679	WD domain-containing protein
NCU04119				x		constitutive	1724	LG V	4908983	4910706	SNARE complex subunit
NCU04149	x	x				constitutive	1896	LG V	5006504	5008399	mitochondrial GTPase
NCU04343	x	x				constitutive	3927	LG IV	3707178	3711104	DUF323 domain-containing protein
NCU04391		x				constitutive	2478	LG IV	3513120	3515597	MFS aflatoxin efflux pump
NCU04421	x					n/a	2255	LG IV	3419585	3421839	annexin 14
NCU04558	x	x				n/a	2013	LG VII	2806028	2808040	hypothetical protein
NCU04628	x	x	x	x		reproductive	3546	LG V	1417820	1421365	hypothetical protein
NCU04645	x	x				reproductive	2150	LG V	1350526	1352675	DUF124 domain-containing protein
NCU04730	x					reproductive	3239	LG VI	574932	578170	post-transcriptional silencing protein QDE-2
NCU04731	x	x		x		n/a	3482	LG VI	581977	585458	HLH transcription factor
NCU04757	x	x				constitutive	3519	LG VI	690842	694360	hypothetical protein
NCU04771			x			constitutive	1960	LG VI	760075	762034	fructosyl-amino acid oxidase
NCU04795	x					constitutive	2142	LG VI	847241	849382	hypothetical protein
NCU04886	x	x	x			constitutive	1854	LG IV	630732	632585	MFS multidrug transporter
NCU04930		x				vegetative	1982	LG IV	777611	779592	hypothetical protein

NCU04994	x					n/a	1371	LG VI	4154030	4155400	leucine aminopeptidase 2
NCU05057		x				reproductive	1389	LG VI	3894456	3895844	endoglucanase EG-1
NCU05191	x	x				reproductive	1364	LG IV	5806268	5807631	hypothetical protein
NCU05271	x					constitutive	4199	LG IV	5510612	5514810	hypothetical protein
NCU05299		x				vegetative	1335	LG IV	5418374	5419708	NADH:ubiquinone oxidoreductase 29.9
NCU05386		x				constitutive	2209	LG II	1483555	1485763	hypothetical protein
NCU05508	x				x	constitutive	1263	LG VI	1679248	1680510	hypothetical protein
NCU05569	x	x				constitutive	807	LG VI	1478617	1479423	hypothetical protein
NCU05585		x				constitutive	2319	LG VI	1422875	1425193	MFS quinate transporter
NCU05735	x	x				constitutive	1997	LG III	679909	681905	membrane transporter
NCU05764	x	x			x	constitutive	1173	LG VII	513445	514617	hypothetical protein
NCU05767	x					n/a	2052	LG VII	497025	499076	PRO1A C6 Zink-finger protein
NCU05861	x	x				reproductive	5000	LG VII	161883	166882	hypothetical protein
NCU05870	x	x				constitutive	1826	LG VII	138805	140630	hypothetical protein
NCU06029	x	x				constitutive	1516	LG VII	2215464	2216979	hypothetical protein
NCU06116	x	x				constitutive	934	LG VII	2503747	2504680	hypothetical protein
NCU06213	x			x		constitutive	2338	LG III	1924734	1927071	MIZ zinc finger protein
NCU06245	x	x				constitutive	2738	LG III	2027776	2030513	phospholipase C-1
NCU06247	x	x	x		x	constitutive	4518	LG III	2033651	2038168	hypothetical protein
NCU06250	x	x				constitutive	2943	LG III	2054183	2057125	F-box/LRR repeat containing protein 2
NCU06256	x	x				constitutive	1874	LG III	2088895	2090768	phosphoglycerate mutase
NCU06353	x		x			constitutive	980	LG IV	2809289	2810268	hypothetical protein
NCU06373	x	x				constitutive	2555	LG IV	2747711	2750265	hypothetical protein
NCU06387	x	x				reproductive	1822	LG IV	2685168	2686989	hypothetical protein
NCU06406	x	x		x	x	constitutive	2913	LG III	1278420	1281332	hypothetical protein
NCU06413	x	x				vegetative	3101	LG III	1314200	1317300	hypothetical protein
NCU06506	x	x			x	constitutive	2533	LG III	1673492	1676024	hypothetical protein
NCU06536	x	x				constitutive	3897	LG IV	1561042	1564938	hypothetical protein
NCU06602	x	x				n/a	1554	LG IV	1771482	1773035	hypothetical protein
NCU06626				x		n/a	5067	LG IV	1845214	1850280	phosphoinositide 3-kinase regulatory subunit 4
NCU06633	x					n/a					
NCU06707	x	x				n/a	2853	LG V	4345793	4348645	hypothetical protein
NCU06842	x					n/a	4250	LG II	1787433	1791682	RCM-1
NCU07003	x	x				constitutive	2384	LG IV	4851982	4854365	hypothetical protein

	dN/dS above average						*N. crassa* annotation information				
Gene	global	*dis*	*int*	*cra*	*tet*	Gene category	Length	LG	Start	Stop	Gene Name
NCU07005	x	x				constitutive	1402	LG IV	4865227	4866628	glycosyl hydrolase
NCU07017	x	x		x		constitutive	1649	LG IV	4920186	4921834	hypothetical protein
NCU07020		x	x			constitutive	2324	LG IV	4931688	4934011	vacuolar protein sorting-associated protein vps17
NCU07022	x					vegetative	1505	LG IV	4941065	4942569	sorbitol dehydrogenase
NCU07063		x				constitutive	1821	LG VI	297396	299216	hypothetical protein
NCU07197					x	n/a	1042	LG V	6270438	6271479	arsenate reductase
NCU07311	x		x	x		reproductive	1353	LG IV	4415379	4416731	hypothetical protein
NCU07352		x				reproductive	1850	LG I	6272683	6274532	hypothetical protein
NCU07368			x			n/a	1577	LG I	6195506	6197082	hypothetical protein
NCU07405	x	x				constitutive	2169	LG I	6048250	6050418	hypothetical protein
NCU07477	x	x		x		constitutive	3871	LG I	494663	498533	hypothetical protein
NCU07622	x	x				constitutive	1953	LG IV	303749	305701	hypothetical protein
NCU07706	x	x				constitutive					
NCU07743	x	x	x			reproductive	2944	LG V	455167	458110	hypothetical protein
NCU07788	x	x				constitutive	2935	LG V	287303	290237	fungal specific transcription factor
NCU07822	x	x	x			n/a	1026	LG III	984188	985213	hypothetical protein
NCU07869		x				constitutive	2479	LG III	1177430	1179908	INSIG domain-containing protein
NCU07883	x	x				constitutive	2404	LG III	1228386	1230789	hypothetical protein
NCU07888	x	x				reproductive	1305	LG III	1240284	1241588	hypothetical protein
NCU07925	x	x				n/a	1549	LG IV	1331443	1332991	LRP16
NCU07974	x	x				constitutive	2217	LG IV	3772956	3775172	endoglucanase B
NCU08136	x	x		x		n/a	3105	LG VII	757665	760769	hypothetical protein
NCU08347		x				constitutive	1848	LG I	5675133	5676980	hypothetical protein
NCU08379	x	x				constitutive	1312	LG I	5552705	5554016	hypothetical protein
NCU08435	x	x	x	x	x	reproductive	4357	LG II	4057221	4061577	RNA-dependent RNA polymerase
NCU08526	x	x				n/a	2205	LG III	5043330	5045534	hypothetical protein
NCU08663	x		x			constitutive	1945	LG IV	4181867	4183811	nonsense-mediated mRNA decay protein 3
NCU08691	x	x				constitutive	1457	LG IV	4082383	4083839	EF-hand calcium-binding domain-containing protein
NCU08719	x					n/a	1761	LG II	3788709	3790469	glutamyl-tRNA(Gln) amidotransferase subunit A
NCU08806	x	x	x			n/a	1756	LG IV	5187850	5189605	hypothetical protein
NCU08903	x	x				constitutive	1339	LG V	695988	697326	Nop10 family nucleolar RNA-binding protein
NCU08977	x	x				constitutive	3005	LG III	4112206	4115210	long chain fatty alcohol oxidase

NCU08986	x	x				reproductive	827	LG III	4138036	4138862	hypothetical protein
NCU09018	x	x				constitutive	1318	LG III	4253696	4255013	glutaminyl-peptide cyclotransferase
NCU09045	x	x	x	x	x	constitutive	2463	LG VII	3819624	3822086	heterokaryon incompatibility protein
NCU09049		x				reproductive	1286	LG VII	3801631	3802916	hypothetical protein
NCU09099	x	x				reproductive	1301	LG I	851721	853021	hypothetical protein
NCU09113	x	x				constitutive					
NCU09133	x	x				vegetative	1825	LG I	5449901	5451725	hypothetical protein
NCU09136	x	x				constitutive	1841	LG I	5459096	5460936	hypothetical protein
NCU09139	x					constitutive	2289	LG I	5465780	5468068	tubulin-specific chaperone
NCU09171		x				reproductive	2248	LG III	241501	243748	hypothetical protein
NCU09207	x	x	x			constitutive	3507	LG I	5838186	5841692	hypothetical protein
NCU09241	x					constitutive	3045	LG I	9439555	9442599	hypothetical protein
NCU09333	x		x			n/a	2946	LG I	4198139	4201084	zinc finger transcription factor ace1
NCU09357			x			reproductive	7454	LG II	4231023	4238476	stage V sporulation protein K
NCU09362	x	x				n/a	3063	LG I	3592036	3595098	hypothetical protein
NCU09365	x	x		x		constitutive	2520	LG I	3583031	3585550	hypothetical protein
NCU09375	x			x		constitutive	1550	LG I	3530171	3531720	hypothetical protein
NCU09466	x	x		x	x	n/a	1723	LG II	4371194	4372916	hypothetical protein
NCU09517					x	vegetative	1351	LG III	35785	37135	hypothetical protein
NCU09575	x	x				reproductive	2407	LG VII	3300200	3302606	sterol esterase
NCU09647		x				n/a	1503	LG IV	59748	61250	hypothetical protein
NCU09656	x	x				n/a	1629	LG IV	85773	87401	carboxymethylenebutenolidase
NCU09715			x			constitutive	2407	LG VII	2875788	2878194	alpha,alpha-trehalose-phosphate synthase
NCU09760	x	x		x		n/a	4436	LG II	4317404	4321839	hypothetical protein
NCU09812	x		x		x	constitutive	2927	LG I	4131500	4134426	LIM domain-containing protein
NCU09881	x	x				constitutive	1841	LG I	6356249	6358089	hypothetical protein
NCU09929	x					constitutive	1690	LG IV	5272027	5273716	hypothetical protein
NCU09994	x	x	x			n/a	2346	LG I	3677688	3680033	hypothetical protein
NCU10034		x				n/a	1201	LG II	3359193	3360393	retrograde vesicle-mediated transporter Get1
NCU10073	x	x				n/a	2855	LG I	4040313	4043167	actin binding protein
NCU10430	x	x				constitutive	1389	LG II	2111818	2113206	SNF7 family protein
NCU10476	x	x		x		vegetative	3323	LG V	3525885	3529207	hypothetical protein
NCU10618				x		n/a	1859	LG V	1565139	1566997	AP-1 complex subunit theta-1
NCU10789	x			x		constitutive	2910	LG I	2760976	2763885	ubiquitin-protein ligase Sel1/Ubx2

Gene	dN/dS above average					Gene category	*N. crassa* annotation information				
	global	*dis*	*int*	*cra*	*tet*		Length	LG	Start	Stop	Gene Name
NCU10997	x	x				vegetative					
NCU11065	x		x			n/a					
NCU11274	x		x			vegetative					
NCU11312	x	x			x	constitutive	2921	LG III	1961297	1964217	CorA family metal ion transporter
NCU11387	x					constitutive					
NCU11388	x		x	x	x	n/a					
