# Supplementary material for: Analyses of expressed sequence tags in Neurospora reveal rapid evolution of genes associated with the early stages of sexual reproduction in fungi
Source: BMC Evol Biol. 2012 Nov 27;12:229. doi: 10.1186/1471-2148-12-229 (PMC3571971; doi:10.1186/1471-2148-12-229)
Supplement: Additional file 2: Table S2 — Candidate genes used to test for positive selection, and ID of additional heterothallic Neurospora strains sequenced. (PDF 51 kb) [file 1471-2148-12-229-S2.pdf]

Supplementary Table 2. Candidate genes used to test for positive selection, and ID of additional heterothallic *Neurospora* strains sequenced. Accession numbers will be given upon acceptance of the manuscript.

		Gene alignment				
Species	Isolate code	NCU01720	NCU03013[1]	NCU03584[2]	NCU06387	NCU07311
N. crassa (NcC)	FGSC 8858	-	-	-	HE861791	-
N. crassa (NcC)	FGSC 8863	HE861780	HE861803	-	-	HE861813
N. crassa (NcB)	FGSC 8772	HE861774	HE861797	HE861766	HE861785	HE861809
N. discreta (PS7)	FGSC 8780	HE861775	HE861798	-	HE861786	-
N. discreta (PS6)	FGSC 8827	HE861777	HE861800	-	HE861788	-
N. hispaniola	FGSC 8815	HE861776	HE861799	HE861767	HE861787	HE861810
N. intermedia (NiA)	FGSC 8901	HE861782	HE861805	HE861770	HE861793	HE861815
N. intermedia (NiB)	FGSC 8768	HE861773	HE861796	-	HE861784	HE861808
N. metzenbergii	FGSC 8853	HE861779	HE861802	-	HE861790	HE861812
N. perkinsii	FGSC 8835	HE861778	HE861801	HE861768	HE861789	HE861811
N. sitophila	FGSC 412	HE861771	HE861794	-	HE861783	HE861806
Genome sequences						
N. crassa[3]		included	included	included	included	included
N. discreta[4]	FGSC 8579	-	-	included	-	included
N. tetrasperma[5]	FGSC 2508	included	included	included	included	included

[1] acw-10 gene for anchored cell wall protein-10
[2] pks gene for polyketide synthase
[3] Neurospora crassa database, Broad Institute (http://www.broadinstitute.org/annotation/genome/neurospora/MultiHome.html)
[4] JGI, Neurospora discreta FGSC 8579 mat A (http://genome.jgi-psf.org/Neudi1/Neudi1.home.html)
[5] JGI, Neurospora tetrasperma FGSC 2508 mat A
