# Supplementary material for: Analyses of expressed sequence tags in Neurospora reveal rapid evolution of genes associated with the early stages of sexual reproduction in fungi
Source: BMC Evol Biol. 2012 Nov 27;12:229. doi: 10.1186/1471-2148-12-229 (PMC3571971; doi:10.1186/1471-2148-12-229)
Supplement: Additional file 3: Figure S1 — Nucleotide and amino acid alignments of the three genes NCU03013, NCU06387, and NCU07311, showing which codons were found to evolve under positive selection. (PDF 13386 kb) [file 1471-2148-12-229-S3.pdf]

```
Gene: NCU03013           intron
                         10        20        30        40        50        60        70        80        90        100       110       120

N_crassa     AAGGCTCTCCCCACAGCTGCAGCTGTGACCAACAATCCCTCTTGTCTCATTGCCGAAGCGGTCCTTCCCGAGAACGCCTGGCAAAAGAACGGATTCCCTAATGGCGGCAACATCAAGGGC
              K  A  L  P  T  A  A  A  V  T  N  N  P  S  C  L  I  A  E  A  V  L  P  E  N  A  W  Q  K  N  G  F  P  N  G  G  N  I  K  G
N_c_B_8772   AAGGCTCTCCCCACTGCTGCAGCTGTGACCAACAATCCGTCTTGTCTCGTTGCCGAGGCGGTCCTTCCCGAGAACGCCTGGCAAAAGAACGGATTCCCTAATGGCGGCAACATCAAGGGC
              K  A  L  P  T  A  A  A  V  T  N  N  P  S  C  L  V  A  E  A  V  L  P  E  N  A  W  Q  K  N  G  F  P  N  G  G  N  I  K  G
N_c_C_8863   AAGGCTCTCCCCACAGCTGCAGCTGTGACCAACAATCCGTCTTGTCTCGTTGCCGAGGCGGTCCTTCCCGAGAACGCCTGGCAAAAGAACGGATTCCCTAATGGCGGCAACATCAAGGGC
              K  A  L  P  T  A  A  A  V  T  N  N  P  S  C  L  V  A  E  A  V  L  P  E  N  A  W  Q  K  N  G  F  P  N  G  G  N  I  K  G
N_dis_8780   AAGCCTCTCCCCACAGCTGCAGCTGTGACCAACAATCCCTCTTGTCTCGTTGCCGAAGCGGTCTTCCCGAGAATGCCTGGCAAAAGAACGGCTTCCCTAATGGCGGCAACATCAAGGGC
              K  P  L  P  T  A  A  A  V  T  N  N  P  S  C  L  V  A  E  A  V  L  P  E  N  A  W  Q  K  N  G  F  P  N  G  G  N  I  K  G
N_dis_8827   AAGCCTCTCCCCACAGCTGCAGCTGTGACCAACAATCCCTCTTGTCTCGTTGCCGAAGCGGTCCTTCCCGAGAACGCCTGGCAAAAGAACGGCTTCCCTAATGGCGGCAACATCAAGGGC
              K  P  L  P  T  A  A  A  V  T  N  N  P  S  C  L  V  A  E  A  V  L  P  E  N  A  W  Q  K  N  G  F  P  N  G  G  N  I  K  G
N_i_A_8901   AAGGCTCTCCCCACAGCTGCAGCTGTGACCAACAACCCCTCTTGTCTCGTTGCCGAAGCGGTCCTTCCCGAGAACGCCTGGCAAAAGAACGGCTTCCCTAATGGCGGCAACATCAAGGGC
              K  A  L  P  T  A  A  A  V  T  N  N  P  S  C  L  V  A  E  A  V  L  P  E  N  A  W  Q  K  N  G  F  P  N  G  G  N  I  K  G
N_i_B_8768   ---GCTCTCCCCACAGCTGCAGCTGTGACCAACAACCCCTCTTGTCTCGTTGCCGAAGCGGTCCTTCCCGAGAACGCCTGGCAAAAGAACGGCTTCCCTAATGGCGGCAACATCAAGGGC
                 A  L  P  T  A  A  A  V  T  N  N  P  S  C  L  V  A  E  A  V  L  P  E  N  A  W  Q  K  N  G  F  P  N  G  G  N  I  K  G
N_his_8815   ------CTCCCCACAGCTGCAGCTGTGACCAACAATCCCTCTTGTCTCGTTGCCGAAGCGGTCCTTCCCGAGAACGCCTGGCAAAAGAACGGCTTCCCTAATGGCGGCAACATCAAGGGC
                    L  P  T  A  A  A  V  T  N  N  P  S  C  L  V  A  E  A  V  L  P  E  N  A  W  Q  K  N  G  F  P  N  G  G  N  I  K  G
N_met_8853   ---CCTCTCCCCACAGCTGCAGCTGTGACCAACAATCCCTCTTGTCTCGTTGCCGAAGCGGTCTTCCCGAGAACGCCTGGCAAAAGAACGGCTTCCCTAATGGCGGCAACATCAAGGGC
                 P  L  P  T  A  A  A  V  T  N  N  P  S  C  L  V  A  E  A  V  L  P  E  N  A  W  Q  K  N  G  F  P  N  G  G  N  I  K  G
N_per_8835   AAGGCTCTCCCCACAGCTGCAGCTGTGACCAACAATCCGTCTTGTCTCGTTGCCGAAGCGGTCCTTCCCGAGAACGCCTGGCAAAAGAACGGATTCCCTAATGGCGGCAACATCAAGGGC
              K  A  L  P  T  A  A  A  V  T  N  N  P  S  C  L  V  A  E  A  V  L  P  E  N  A  W  Q  K  N  G  F  P  N  G  G  N  I  K  G
N_sit_0412   ACGGCTCTCCCCACAGCTGCAGCTGTGACCAACAATCCCTCTTGTCTCGTTGCCGAAGCGGTCCTTCCCGAGAACGCCTGGCAAAAGAACGGCTTCCCTAATGGTGGCAACATCCAGGGC
              T  A  L  P  T  A  A  A  V  T  N  N  P  S  C  L  V  A  E  A  V  L  P  E  N  A  W  Q  K  N  G  F  P  N  G  G  N  I  Q  G
N_tetraspe   AAGGCTCTCCCCACAGCTGCAGCTGTGACCAACAATCCCTCTTGTCTCGTTGCCGAAGCGGTCCTTCCAGAAAACGCCTGGCAAAAGAACGGCTTCCCTAATGGCGGCAACATCAAGGGC
              K  A  L  P  T  A  A  A  V  T  N  N  P  S  C  L  V  A  E  A  V  L  P  E  N  A  W  Q  K  N  G  F  P  N  G  G  N  I  K  G

                       130       140       150       160       170       180       190       200       210       220       230       240

N_crassa     AAGGTGGTAGCCAAGTCGGGCGATGGCGGTGTTGGAGTGCAGTTCAATGTTGAAGTGAGCGGCCTGCCTGAGGGTGGCCCTTTCACTTACCACATTCACGCCAAACCCGTCCCCGAGAAC
              K  V  V  A  K  S  G  D  G  G  V  G  V  Q  F  N  V  E  V  S  G  L  P  E  G  G  P  F  T  Y  H  I  H  A  K  P  V  P  E  N
N_c_B_8772   AAGGTAGTTGCCAAGTCGGGCGATGGCGGTGTTGGAGTGCAGTTCAATGTTGAAGTGAGCGGCCTGCCTGAAGGTGGCCCTTTCACTTACCACATTCACGCCAAACCCGTCCCCGAGAAC
              K  V  V  A  K  S  G  D  G  G  V  G  V  Q  F  N  V  E  V  S  G  L  P  E  G  G  P  F  T  Y  H  I  H  A  K  P  V  P  E  N
N_c_C_8863   AAGGTGGTAGCCAAGTCGGGCGATGGCGGTGTTGGAGTGCAGTTCAATGTTGAAGTGAGCGGCCTGCCTGAGGGTGGCCCTTTCACTTACCACATTCACGCCAAGCCCGTCCCCGAGAAC
              K  V  V  A  K  S  G  D  G  G  V  G  V  Q  F  N  V  E  V  S  G  L  P  E  G  G  P  F  T  Y  H  I  H  A  K  P  V  P  E  N
N_dis_8780   AAGGTGGTAGCCAAGTCGGGCGAGGGCGGTGTTGGAGTGCAGTTCAATGTTGAAGTGAGCGGCCTGCCTGAGGGTGGCCCTTTCACTTACCACATTCACGCCAAACCCGTCCCCGAGAAC
              K  V  V  A  K  S  G  E  G  G  V  G  V  Q  F  N  V  E  V  S  G  L  P  E  G  G  P  F  T  Y  H  I  H  A  K  P  V  P  E  N
N_dis_8827   AAGGTGGTAGCCAAGTCGGGCGAGGGCGGTGTTGGAGTGCAGTTCAATGTTGAAGTGAGCGGCCTGCCTGAGGGTGGCCCTTTCACTTACCACATTCACGCCAAACCCGTCCCCGAGAAC
              K  V  V  A  K  S  G  E  G  G  V  G  V  Q  F  N  V  E  V  S  G  L  P  E  G  G  P  F  T  Y  H  I  H  A  K  P  V  P  E  N
N_i_A_8901   AAGGTGGTAGCCGAGTCGGGCGACGGCGGTGTTGGAGTGCAGTTCAATGTTGAAGTGAGCGGCCTGCCTGAGGGTGGCCCTTTCACTTACCACATTCACGCCAAGCCCGTCCCCGAGAAC
              K  V  V  A  E  S  G  D  G  G  V  G  V  Q  F  N  V  E  V  S  G  L  P  E  G  G  P  F  T  Y  H  I  H  A  K  P  V  P  E  N
N_i_B_8768   AAGGTGGTAGCCAAGTCGGGCGACGGCGGTGTTGGAGTGCAGTTCAATGTTGAAGTGAGCGGCCTGCCTGAGGGTGGCCCTTTCACTTACCACATTCACGCCAAGCCCGTCCCCGAGAAC
              K  V  V  A  K  S  G  D  G  G  V  G  V  Q  F  N  V  E  V  S  G  L  P  E  G  G  P  F  T  Y  H  I  H  A  K  P  V  P  E  N
N_his_8815   AAGGTGGTAGCCAAGTCGGGCGACGGCGGTGTTGGAGTGCAGTTCAATGTTGAAGTGAGCGGCCTGCCTGAGGGTGGCCCTTTCACTTACCACATTCACGCCAAACCCGTCCCCGAGAAC
              K  V  V  A  K  S  G  D  G  G  V  G  V  Q  F  N  V  E  V  S  G  L  P  E  G  G  P  F  T  Y  H  I  H  A  K  P  V  P  E  N
N_met_8853   AAGGTGGTAGCCAAGTCGGGCGACGGCGGTGTTGGAGTGCAGTTCAATGTTGAAGTGAGCGGCCTGCCTGAGGGTGGCCCTTTCACTTACCACATTCACGCCAAACCCGTCCCCGAGAAC
              K  V  V  A  K  S  G  D  G  G  V  G  V  Q  F  N  V  E  V  S  G  L  P  E  G  G  P  F  T  Y  H  I  H  A  K  P  V  P  E  N
N_per_8835   AAGGTGGTAGCCAAGTCGGGCGATGGCGGTGTTGGAGTGCAGTTCAATGTTGAAGTGAGCGGCCTGCCTGAGGGTGGCCCTTTCACTTACCACATTCACGCCAAACCCGTCCCCGAGAAC
              K  V  V  A  K  S  G  D  G  G  V  G  V  Q  F  N  V  E  V  S  G  L  P  E  G  G  P  F  T  Y  H  I  H  A  K  P  V  P  E  N
N_sit_0412   AAGGTAGTAGCCAAGTCGGGCGATGGCGGCGTTGGAGTGCAGTTCAATGTTGAAGTGAGCGGCCTGCCTGAGGGTGGCCCTTTCACTTACCACATTCACGCCAAACCCGTCCCCGAGAAC
              K  V  V  A  K  S  G  D  G  G  V  G  V  Q  F  N  V  E  V  S  G  L  P  E  G  G  P  F  T  Y  H  I  H  A  K  P  V  P  E  N
N_tetraspe   AAGGTGGTAGCCAAGTCGGGCGACGGCGGTGTTGGAGTGCAGTTCAACGTTGAAGTGAGCGGCCTGCCTGAGGGTGGCCCTTTCACTTACCACATTCACGCCAAGCCCGTCCCCGAGAAC
              K  V  V  A  K  S  G  D  G  G  V  G  V  Q  F  N  V  E  V  S  G  L  P  E  G  G  P  F  T  Y  H  I  H  A  K  P  V  P  E  N
                                                                                                intron
```

```
                   250       260       270       280       290       300       310       320       330       340       350       360
               ....|....|....|....|....|....|....|....|....|....|....|....|....|....|....|....|....|....|....|....|....|....|....|....|
N_crassa       GGCAACTGCACAGCAACCGGCGCTCACTTTGACCCTACTGAGCGAGGCGAGGACCCCGCCTGCGACAAGAGCAAGCCCGAGACGTGCCAGATCGGCGATCTGGCCGGCAAGCACGGCGCT
                G  N  C  T  A  T  G  A  H  F  D  P  T  E  R  G  E  D  P  A  C  D  K  S  K  P  E  T  C  Q  I  G  D  L  A  G  K  H  G  A
N_c_B_8772     GGCAACTGCACAGCAACCGGCGCTCACTTTGACCCTACTGAGCGAGGCGAGGATCCCGCCTGCGACAAGAGCAAGCCCGAGACGTGCCAGATCGGTGATCTGGCCGGCAAGCACGGCGCT
                G  N  C  T  A  T  G  A  H  F  D  P  T  E  R  G  E  D  P  A  C  D  K  S  K  P  E  T  C  Q  I  G  D  L  A  G  K  H  G  A
N_c_C_8863     GGCAACTGCACAGCAACCGGCGCTCACTTTGACCCTACTGAGCGAGGCGAGGACCCCGCCTGCGACAAGAGCAAGCCCGAGACGTGCCAGATCGGCGATCTGGCCGGCAAGCACGGCGCT
                G  N  C  T  A  T  G  A  H  F  D  P  T  E  R  G  E  D  P  A  C  D  K  S  K  P  E  T  C  Q  I  G  D  L  A  G  K  H  G  A
N_dis_8780     GGCAACTGCACGGCAACCGGCGCTCATTTTGACCCTACTGAGCGAGGCGAGGACCCCGTCTGCGACAAGAGCAAGCCCGAGACGTGCCAGATCGGCGATCTGGCCGGCAAGTACGGCGCT
                G  N  C  T  A  T  G  A  H  F  D  P  T  E  R  G  E  D  P  V  C  D  K  S  K  P  E  T  C  Q  I  G  D  L  A  G  K  Y  G  A
N_dis_8827     GGCAACTGCACGGCAACCGGCGCTCATCTTGACCCTACTGAGCGAGGCGAGGACCCCGTCTGCGACAAGAGCAAGCCCGAGACGTGCCAGATCGGCGATCTGGCCGGCAAGTACGGCGCT
                G  N  C  T  A  T  G  A  H  L  D  P  T  E  R  G  E  D  P  V  C  D  K  S  K  P  E  T  C  Q  I  G  D  L  A  G  K  Y  G  A
N_i_A_8901     GGCAACTGCACAGCAACCGGCGCTCACTTTGACCCTACTGAGCGAGGCGAGGACCCCGTCTGCGACAAGAGCAAGCCCGAGACGTGCCAAATCGGCGATCTGGCCGGAAAGCACGGCGCT
                G  N  C  T  A  T  G  A  H  F  D  P  T  E  R  G  E  D  P  V  C  D  K  S  K  P  E  T  C  Q  I  G  D  L  A  G  K  H  G  A
N_i_B_8768     GGCAACTGCACAGCAACCGGCGCTCACTTTGACCCTACTGAGCGAGGCGAGGACCCCGCCTGCGACAAGAGTAAGCCCGAGACGTGCCAGATCGGCGATCTGGCCGGCAAGCACGGCGCT
                G  N  C  T  A  T  G  A  H  F  D  P  T  E  R  G  E  D  P  A  C  D  K  S  K  P  E  T  C  Q  I  G  D  L  A  G  K  H  G  A
N_his_8815     GGCAACTGCACAGCAACCGGCGCTCACTTTGACCCTACTGAGCGAGGCGAGGACCCCGTCTGCGACAAGAGTAAGCCCGAGACGTGCCAGATCGGCGATCTGGCCGGCAAGCACGGCGCT
                G  N  C  T  A  T  G  A  H  F  D  P  T  E  R  G  E  D  P  V  C  D  K  S  K  P  E  T  C  Q  I  G  D  L  A  G  K  H  G  A
N_met_8853     GGCAACTGCACAGCAACCGGCGCTCACTTTGATCCTACTGAGCGAGGCGAGGACCCCGTCTGCGACAAGAGTAAGCCCGAGACGTGCCAGATCGGCGATCTGGCTGGCAAGCACGGCGCT
                G  N  C  T  A  T  G  A  H  F  D  P  T  E  R  G  E  D  P  V  C  D  K  S  K  P  E  T  C  Q  I  G  D  L  A  G  K  H  G  A
N_per_8835     GGCAACTGCACAGCAACCGGCGCTCACTTTGACCCTACTGAGCGAGGCGAGGACCCCGCCTGCGACAAGAGCAAGCCCGAGACGTGCCAGATCGGCGATCTGGCCGGCAAGCACGGCGCT
                G  N  C  T  A  T  G  A  H  F  D  P  T  E  R  G  E  D  P  A  C  D  K  S  K  P  E  T  C  Q  I  G  D  L  A  G  K  H  G  A
N_sit_0412     GGCAACTGCACAGCAACCGGCGCTCACTTTGACCCTACTGAGCGAGGCGAGGACCCCGTCTGCGACAAGAGTAAGCCCGAGACGTGCCAGATCGGCGATCTTGCCGGCAAGTACGGCGCT
                G  N  C  T  A  T  G  A  H  F  D  P  T  E  R  G  E  D  P  V  C  D  K  S  K  P  E  T  C  Q  I  G  D  L  A  G  K  Y  G  A
N_tetraspe     GGCAACTGCACAGCAACCGGCGCTCACTTTGACCCTACTGAGCGAGGCGAGGACCCCGTCTGCGACAAGAGTAAGCCCGAGACGTGCCAGATCGGCGATCTGGCTGGCAAGCACGGCGCT
                G  N  C  T  A  T  G  A  H  F  D  P  T  E  R  G  E  D  P  V  C  D  K  S  K  P  E  T  C  Q  I  G  D  L  A  G  K  H  G  A

                   370       380       390       400       410       420       430       440       450       460       470       480
               ....|....|....|....|....|....|....|....|....|....|....|....|....|....|....|....|....|....|....|....|....|....|....|....|
N_crassa       ATTCCTGCGGGCAACACCACCTTCAGCGCCAGTTACGTTGACAAGTACGCCTCGCTCGTTGAGGGTTCAGACGCCTACTTCCTCGACCGCAGTATCGTTTTCCATTTCCCCAACAAGACG
                I  P  A  G  N  T  T  F  S  A  S  Y  V  D  K  Y  A  S  L  V  E  G  S  D  A  Y  F  L  D  R  S  I  V  F  H  F  P  N  K  T
N_c_B_8772     ATTCCTGCGGGTAATACCACCTTCAGCGCCAGTTACGTTGACAAGTACGCCTCGCTCGTTGAGGGTTCGGACGCCTACTTCCTCGACCGCAGTATCGTGTTCCATTTCCCCAACAAGACG
                I  P  A  G  N  T  T  F  S  A  S  Y  V  D  K  Y  A  S  L  V  E  G  S  D  A  Y  F  L  D  R  S  I  V  F  H  F  P  N  K  T
N_c_C_8863     ATTCCTGCGGGCAACACCACCTTCAGCGCCAGTTACGTTGACAAGTACGCCTCGCTCGTTGAGGGTTCGGATGCCTACTTCCTCGACCGCAGCATCGTGTTCCATTTCCCCAACAAGACG
                I  P  A  G  N  T  T  F  S  A  S  Y  V  D  K  Y  A  S  L  V  E  G  S  D  A  Y  F  L  D  R  S  I  V  F  H  F  P  N  K  T
N_dis_8780     ATTCCTGCGGGCAATACCACCTTCAGCGCCAATTACGTTGACAAGTACACCTCGCTCGTTGAGGGTACGAGCGCCTATTTCCTCGACCGCAGCATCGTGTTCCATTTCCCCAACAAGACG
                I  P  A  G  N  T  T  F  S  A  N  Y  V  D  K  Y  T  S  L  V  E  G  T  S  A  Y  F  L  D  R  S  I  V  F  H  F  P  N  K  T
N_dis_8827     ATTCCTGCGGGCAATACCACCTTCAGCGCCAATTACGTTGACAAGTACACCTCGCTCGTTGAGGGTACGAGCGCCTACTTCCTCGACCGCAGCATCGTGTTCCATTTCCCCAACAAGACG
                I  P  A  G  N  T  T  F  S  A  N  Y  V  D  K  Y  T  S  L  V  E  G  T  S  A  Y  F  L  D  R  S  I  V  F  H  F  P  N  K  T
N_i_A_8901     ATTCCTGCGGACAATACCACCTTCAGTGCCAGTTACGTTGACAAGTACGCCTCGCTCGTTGAGGGTTCGGACGCCTACTTCCTCGACCGCAGCATCGTGTTCCATTTCCCCAACAAGACG
                I  P  A  D  N  T  T  F  S  A  S  Y  V  D  K  Y  A  S  L  V  E  G  S  D  A  Y  F  L  D  R  S  I  V  F  H  F  P  N  K  T
N_i_B_8768     ATTCCTGCGGACAATACCACCTTCAGCGCCAGTTACGTTGACAAGTACGCCTCGCTCGTTGAGGGTTCGGACGCCTACTTCCTCGACCGCAGCATCGTGTTCCATTTCCCCAACAAGACG
                I  P  A  D  N  T  T  F  S  A  S  Y  V  D  K  Y  A  S  L  V  E  G  S  D  A  Y  F  L  D  R  S  I  V  F  H  F  P  N  K  T
N_his_8815     ATTCCTGCGGGCAATACCACCTTCAGCGCCAGTTACGTTGACAAGTACGCCTCACTCGTTGAGGGTTCGGATGCCTACTTCCTCGACCGCAGCATCGTGTTCCATTTCCCCAACAAGACG
                I  P  A  G  N  T  T  F  S  A  S  Y  V  D  K  Y  A  S  L  V  E  G  S  D  A  Y  F  L  D  R  S  I  V  F  H  F  P  N  K  T
N_met_8853     ATTCCTGCGGACAATACCACCTTCAGCGCCAGTTACGTTGACAAGTACGCCTCGCTCGTTGAGGGTTCGGACGCCTACTTCCTCGACCGCAGCATCGTGTTCCATTTCCCCAACAAGACG
                I  P  A  D  N  T  T  F  S  A  S  Y  V  D  K  Y  A  S  L  V  E  G  S  D  A  Y  F  L  D  R  S  I  V  F  H  F  P  N  K  T
N_per_8835     ATTCCTGCGGGCAACACCACCTTCAGCGCCAGTTACGTTGACAAGTACGCCTCGCTCGTTGAGGGTTCGGACGCCTACTTCCTCGACCGCAGCATCGTGTTCCATTTCCCCAACAAGACG
                I  P  A  G  N  T  T  F  S  A  S  Y  V  D  K  Y  A  S  L  V  E  G  S  D  A  Y  F  L  D  R  S  I  V  F  H  F  P  N  K  T
N_sit_0412     ATTCCTGCGGACAATACCACCTTCAGCGCCAGTTACGTTGACAAGTACGCCTCGCTCGTTGAGGGTTCGGACACCTACTTCCTCGACCGCAGCATCGTGTTCCATTTCCCCAACAAGACG
                I  P  A  D  N  T  T  F  S  A  S  Y  V  D  K  Y  A  S  L  V  E  G  S  D  T  Y  F  L  D  R  S  I  V  F  H  F  P  N  K  T
N_tetraspe     ATTCCTGCGGACAATATCACCTTCAGCGCCAGTTACGTTGACAAGTACGCCTCGCTCGTTGAGGGTTCGGACGCCTACTTCCTCGACCGCAGCATCGTGTTCCATTTCCCCAACAAGACG
                I  P  A  D  N  I  T  F  S  A  S  Y  V  D  K  Y  A  S  L  V  E  G  S  D  A  Y  F  L  D  R  S  I  V  F  H  F  P  N  K  T
```

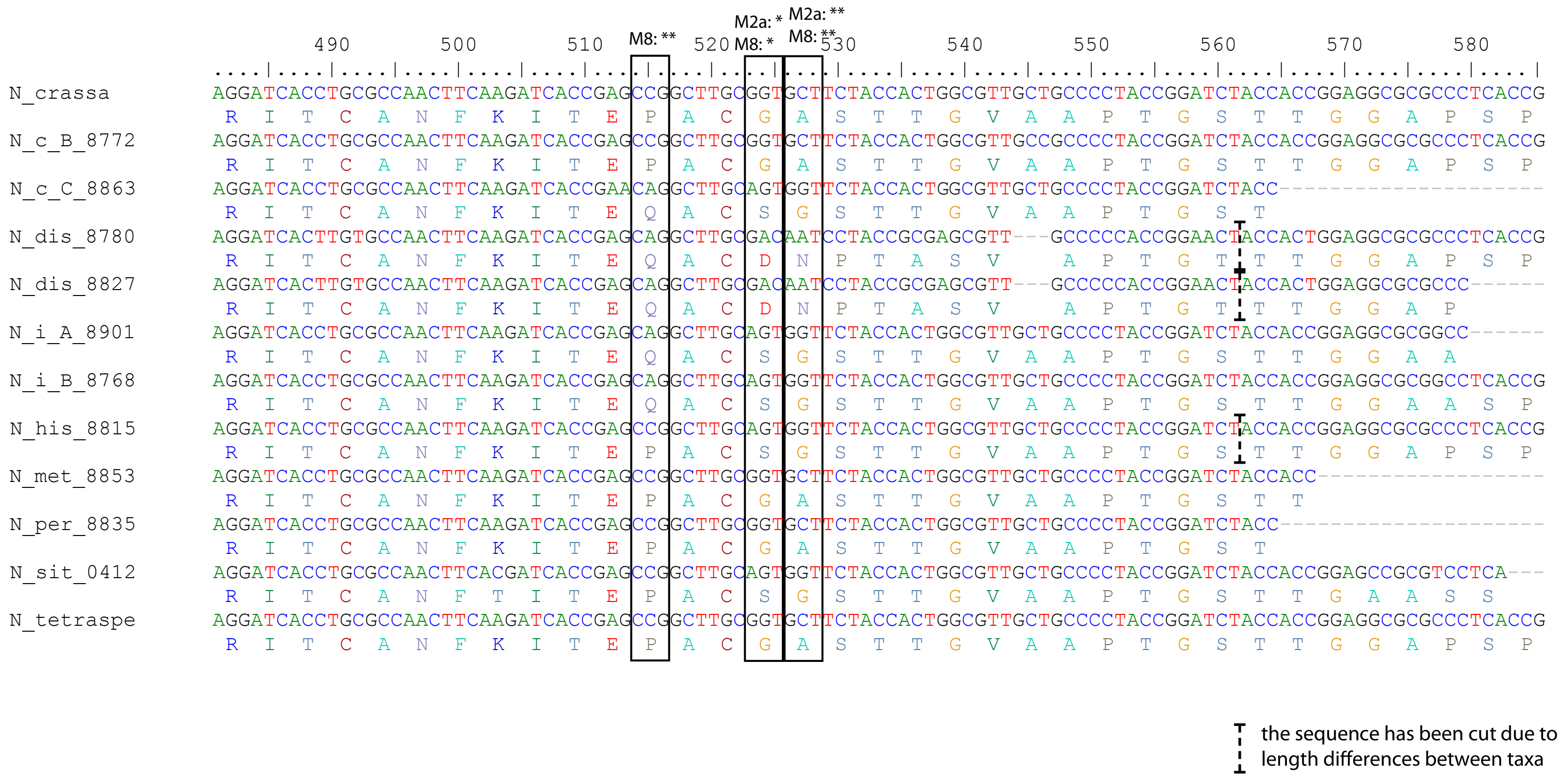


Gene: NCU03013

Compared to the annotated sequence for N. crassa, the alignment is missing the first 20 codons and the last 35 codons.

The sequence N_crassa is the annotated gene sequence for NCU03013.

The sequence N_tetraspe is the gene sequence from the N. tetrasperma genome sequence.

Gene: NCU06387

```
                    10        20        30        40        50        60        70        80        90       100       110       120
            ....|....|....|....|....|....|....|....|....|....|....|....|....|....|....|....|....|....|....|....|....|....|....|....|
N_crassa    AGGCATGCCGCCCTCGATGCTCTCATGGGTGGTCCAACAGGCTGGAAGGAGGCCGATCGACTGAGAATCATGGAGGAGAACCGAAGGAGGAGTATCATGACAGCAAACGGCATGGGTATG
             R  H  A  A  L  D  A  L  M  G  G  P  T  G  W  K  E  A  D  R  L  R  I  M  E  E  N  R  R  R  S  I  M  T  A  N  G  M  G  M
N_c_B_8772  ------------------------------------------------------------------------------------------------------------------------

N_c_C_8858  AGGCATGCCGCCCTCGATGCTCTCATGGGTGGTCCAACGGGCTGGAAGGAGGCCGATCGACTCAGAATCATGGAGGAGAACCGAAGGAGGAGTGTCATGACAGCAAACGGCATGGGTATG
             R  H  A  A  L  D  A  L  M  G  G  P  T  G  W  K  E  A  D  R  L  R  I  M  E  E  N  R  R  R  S  V  M  T  A  N  G  M  G  M
N_dis_8780  ------------------------------------------------GAGGCCGATCGACTCAGGATCATGGAGGAGAACCGAAGGAGGGGTACCATGACAGCAAATGGCATGGGCATG
                                                             E  A  D  R  L  R  I  M  E  E  N  R  R  R  G  T  M  T  A  N  G  M  G  M
N_dis_8827  ------------------GCTCTAATGGGTGGTCCGACGGGCTGGAAGGAGGCCGATCGACTCAGAATCATGGAGGAGAACCGAAGGAGGAGTGCCATGACAGCAAATGGCATGGGCATG
                               A  L  M  G  G  P  T  G  W  K  E  A  D  R  L  R  I  M  E  E  N  R  R  R  S  A  M  T  A  N  G  M  G  M
N_his_8815  AGGCATGCCGCCCTCGATGCTCTCATGGGTGGTCCAACGGGCTGGAAGGAGGCCGATCGACTCAGAATCATGGAGGAGAACCGAAGGAGGAGTGTCATGACAGCAAACGGCATGGGTATG
             R  H  A  A  L  D  A  L  M  G  G  P  T  G  W  K  E  A  D  R  L  R  I  M  E  E  N  R  R  R  S  V  M  T  A  N  G  M  G  M
N_i_A_8901  ------------------------------------------------------------------------------------------------------------------------

N_i_B_8768  AGGCATGCCGCTCTCGATGCTCTCATGGGTGGTCCAACGGGCTGGAAGGAGGCCGATAGACTCAGAATCATGGAGGAGAACCGAAGGAGGAGTGTCATGACGGCAAACGGCATGGGTATG
             R  H  A  A  L  D  A  L  M  G  G  P  T  G  W  K  E  A  D  R  L  R  I  M  E  E  N  R  R  R  S  V  M  T  A  N  G  M  G  M
N_met_8853  ------------------------------------------------------------------------------------AGGAGGAGTGTCATGACAGCAAACGGCATGGGTATG
                                                                                                 R  R  S  V  M  T  A  N  G  M  G  M
N_per_8835  AGGCATGCCGCCCTCGATGCTCTCATGGGTGGTCCAACGGGCTGGAAGGAGGCCGATCGACTCAGAATCATGGAGGAGAACCGAAGGAGGAGTGTCATGACAGCAAACGGCATGGGTATG
             R  H  A  A  L  D  A  L  M  G  G  P  T  G  W  K  E  A  D  R  L  R  I  M  E  E  N  R  R  R  S  V  M  T  A  N  G  M  G  M
N_sit_0412  AGGCATGCCGCCCTCGATGCTCTCATGGGTGGTCCAACGGGCTGGAAGGAGGCCGATCGACTCAGAATCATGGAGGAGAACCGAAGGAGGAGTGTCATGACAGCAAACGGCATGGGTATG
             R  H  A  A  L  D  A  L  M  G  G  P  T  G  W  K  E  A  D  R  L  R  I  M  E  E  N  R  R  R  S  V  M  T  A  N  G  M  G  M
N_tetraspe  AGGCATGCCGCCCTCGATGCTCTCATGGGTGGTCCAACGGGCTGGAAGGAGGCCGATCGACTCAGAATCATGGAGGAGAACCGAAGGAGGAGTGTCATGACAGCAAACGGCATGGGTATG
             R  H  A  A  L  D  A  L  M  G  G  P  T  G  W  K  E  A  D  R  L  R  I  M  E  E  N  R  R  R  S  V  M  T  A  N  G  M  G  M

                   130       140       150       160       170       180       190       200       210       220       230       240
            ....|....|....|....|....|....|....|....|....|....|....|....|....|....|....|....|....|....|....|....|....|....|....|....|
N_crassa    CCGAGCGGACTTTCAACTCCAGTCCACGCCGGTGTCTTTACGCGCGCCAACAGCAGCCTCACGCACGTTTCATACAACAACCCCAGCGGCTACGCCACACCCGTCCCTCCAATTCCCCGA
             P  S  G  L  S  T  P  V  H  A  G  V  F  T  R  A  N  S  S  L  T  H  V  S  Y  N  N  P  S  G  Y  A  T  P  V  P  P  I  P  R
N_c_B_8772  ------------------------------------------------------------------------------------------------------------------------

N_c_C_8858  CCGAGCGGACTGTCAACTCCAGTCCACGCCGGTGTCTTTACGCGCGCCAACAGCAGCCTCACGCACGTTTCATACAACAACCCCAGCGGCTACGCCACACCCGTCCCTCCAATTCCCCGA
             P  S  G  L  S  T  P  V  H  A  G  V  F  T  R  A  N  S  S  L  T  H  V  S  Y  N  N  P  S  G  Y  A  T  P  V  P  P  I  P  R
N_dis_8780  CCGAGCGGAGTTTCAACTCCAGTCCACGCCGGTGTCTTTACCCGCGCCAACAGCAGCCTCACGCATGTTTCATTCAGCAACCCCAGCGGCTACGCAACACCCGTCCCTCCAATTCCCCGA
             P  S  G  V  S  T  P  V  H  A  G  V  F  T  R  A  N  S  S  L  T  H  V  S  F  S  N  P  S  G  Y  A  T  P  V  P  P  I  P  R
N_dis_8827  CCGAGCGGACTTTCAACTCCAGTCCACGCCGGTGTCTTTACCCGCGCCAACAGCAGCCTCACACATGTTTCATTCAGCAACCCCAGCGGCTACGCAACACCCGTCCCTCCAATTCCCCGA
             P  S  G  L  S  T  P  V  H  A  G  V  F  T  R  A  N  S  S  L  T  H  V  S  F  S  N  P  S  G  Y  A  T  P  V  P  P  I  P  R
N_his_8815  CCGAGCGGACTCTCAACTCCAGTCCACGCCGGTGTCTTTACGCGCGCCAACAGCAGCCTCACGCACGTTTCATACAACAACCCCAGCGGCTACGCCACACCCGTCCCTCCAATTCCCCGA
             P  S  G  L  S  T  P  V  H  A  G  V  F  T  R  A  N  S  S  L  T  H  V  S  Y  N  N  P  S  G  Y  A  T  P  V  P  P  I  P  R
N_i_A_8901  ---------------------------------------------------------------------------------------------------CCCGTCCCTCCGATTCCCCGA
                                                                                                          P  V  P  P  I  P  R
N_i_B_8768  CCGAGCGGACTCTCAACTCCAGTCCACGCCGGTGTCTTTACGCGCGCCAACAGCAGCCTCACGCACGTTTCATTCAACAACCCCAGCGGCTCCGTCACACCCGTCCCTCCGATTCCCCGA
             P  S  G  L  S  T  P  V  H  A  G  V  F  T  R  A  N  S  S  L  T  H  V  S  F  N  N  P  S  G  S  V  T  P  V  P  P  I  P  R
N_met_8853  CCGAGCGGACTGTCAACTCCAGTCCACGCCGGTGTCTTTACGCGCGCCAACAGCAGCCTCACGCACGTTTCATACAACAACCCCAGCGGCTACGCCACACCCGTCCCTCCAATTCCCCGA
             P  S  G  L  S  T  P  V  H  A  G  V  F  T  R  A  N  S  S  L  T  H  V  S  Y  N  N  P  S  G  Y  A  T  P  V  P  P  I  P  R
N_per_8835  CCGAGCGGACTGTCAACTCCAGTCCACGCCGGTGTCTTTACGCGCGCCAACAGCAGCCTCACGCACGTTTCATACAACAACCCCAGCGGCTACGCCACACCCGTCCCTCCAATTCCCCGA
             P  S  G  L  S  T  P  V  H  A  G  V  F  T  R  A  N  S  S  L  T  H  V  S  Y  N  N  P  S  G  Y  A  T  P  V  P  P  I  P  R
N_sit_0412  CCGAGCGGACTGTCAACTCCAGTCCACGCCGGTGTCTTTACGCGCGCCAACAGCAGCCTCACGCACGTTTCATCCAACAACCCCAGCGGCTACGCCACACCCGTCCCTCCAATTCCCCGA
             P  S  G  L  S  T  P  V  H  A  G  V  F  T  R  A  N  S  S  L  T  H  V  S  S  N  N  P  S  G  Y  A  T  P  V  P  P  I  P  R
N_tetraspe  CCGAGCGGACTCTCAACTCCAGTCCACGCCGGTGTCTTTACGCGCGCCAACAGCAGCCTCACGCACGTTTCATATAACAACCCCAGCGGCTACGCCACACCCGTCCCTCCAATTCCCCGA
             P  S  G  L  S  T  P  V  H  A  G  V  F  T  R  A  N  S  S  L  T  H  V  S  Y  N  N  P  S  G  Y  A  T  P  V  P  P  I  P  R
```

```
                250       260       270       280       290       300       310       320       330       340       350       360
             ....|....|....|....|....|....|....|....|....|....|....|....|....|....|....|....|....|....|....|....|....|....|....|....|
N_crassa     GCATACAGCTACCACGGCTCGGTCCCGGGGTGGAGCCATCATGGCGGTGAAGTCAGCTATCTCTCGATCGATATGGCTGGAGCTGCCAGCAGTGGAACATCGATCAAGGGAAAAGAAAAG
              A  Y  S  Y  H  G  S  V  P  G  W  S  H  H  G  G  E  V  S  Y  L  S  I  D  M  A  G  A  A  S  S  G  T  S  I  K  G  K  E  K
N_c_B_8772   ---------------GGCTCGGTCCCGGGGTGGAGCCATCATGGCGGTGAAGTCAGCTATCTCTCAATCGATATGGCTGGAGCTGCCAGCAGTGGAACATCGATTAAGGGAAAAGAAAAG
                             G  S  V  P  G  W  S  H  H  G  G  E  V  S  Y  L  S  I  D  M  A  G  A  A  S  S  G  T  S  I  K  G  K  E  K
N_c_C_8858   GCATACAGCTACCACGGCTCGGTCCCGGGGTGGAGCCATCATGGCGGTGAAGTCAGCTATCTCTCGATCGATATGGCTGGAGCTGCCAGCAGCGGAACATCGATAAAGGGAAAAGAAAAG
              A  Y  S  Y  H  G  S  V  P  G  W  S  H  H  G  G  E  V  S  Y  L  S  I  D  M  A  G  A  A  S  S  G  T  S  I  K  G  K  E  K
N_dis_8780   GCATACAGCTACCACGGCCCGGTCCCGGGGTGGAGCCATCATGGCGGTGAGATCAGCTATCTCTCAATCGACATGGCGGGAGCTGCCAGCAGTGGAACATCGATAAAGGGGAAAGAGAAA
              A  Y  S  Y  H  G  P  V  P  G  W  S  H  H  G  G  E  I  S  Y  L  S  I  D  M  A  G  A  A  S  S  G  T  S  I  K  G  K  E  K
N_dis_8827   GCATACAGCTACCACGGCCCGGTCCCGGGGTGGAGCCATCATGGCGGTGAGATCAGCTATCTCTCAATCGACATGGCGGGAGCCGCCAGCAGTGGAACATCGATAAAGGGGAAAGAGAAA
              A  Y  S  Y  H  G  P  V  P  G  W  S  H  H  G  G  E  I  S  Y  L  S  I  D  M  A  G  A  A  S  S  G  T  S  I  K  G  K  E  K
N_his_8815   GCATACAGCTACCACGGCTCGATCCCGGGATGGAGCCATCATGGCGGTGAAGTCAGCTATCTCTCGATCGACATGGCTGGAGCTGCCAGCAGTGGAACATCGATCAAGGGAAAAGAAAAG
              A  Y  S  Y  H  G  S  I  P  G  W  S  H  H  G  G  E  V  S  Y  L  S  I  D  M  A  G  A  A  S  S  G  T  S  I  K  G  K  E  K
N_i_A_8901   GCATACAGCTACCACGGCTCAGTCCCGGGGTGGAGCCATCATGGCGGTGAAGTCAGCTATCTCTCTATCGATATGGCTGGAGCTGCCAGCAGTGGAACATCGATAAAGGGAAAAGAGAAG
              A  Y  S  Y  H  G  S  V  P  G  W  S  H  H  G  G  E  V  S  Y  L  S  I  D  M  A  G  A  A  S  S  G  T  S  I  K  G  K  E  K
N_i_B_8768   GCATACAGCTACCACGGCTCAGTCCCGGGGTGGAGCCATCATGGCGGTGAAGTCAGCTATCTCTCGATCGATATGGCTGGAGCTGCCAGCAGTGGAACATCGATAAAGGGAAAAGAGAAG
              A  Y  S  Y  H  G  S  V  P  G  W  S  H  H  G  G  E  V  S  Y  L  S  I  D  M  A  G  A  A  S  S  G  T  S  I  K  G  K  E  K
N_met_8853   GCATACAGCTACCACGGCTCGGTCCCGGGGTGGAGCTATCATGGCGGTGAAGTCAGCTATCTCTCGATCGATATGGCTGGAGCTGCCAGCAGTGGAACATCGATAAAGGGAAAAGAAAAG
              A  Y  S  Y  H  G  S  V  P  G  W  S  Y  H  G  G  E  V  S  Y  L  S  I  D  M  A  G  A  A  S  S  G  T  S  I  K  G  K  E  K
N_per_8835   GCATACAGCTACCACGGCTCGGTCCCGGGGTGGACCCATCATGGCGGTGAAGTCAGCTATCTCTCGATCGATATGGCTGGAGCTGTCAGCAGTGGAACATCGATAAAGGGAAAAGAAAAG
              A  Y  S  Y  H  G  S  V  P  G  W  T  H  H  G  G  E  V  S  Y  L  S  I  D  M  A  G  A  V  S  S  G  T  S  I  K  G  K  E  K
N_sit_0412   GCATACAGCTACCACGGCTCGGTTCCGGGTTGGAGCCATCATGGCGGTGAAGTCAGCTATCTCTCGATCGATATGGCTGGAGCTGCCAGCAGTGGAACATCGATAAAGGGAAAAGAAAAG
              A  Y  S  Y  H  G  S  V  P  G  W  S  H  H  G  G  E  V  S  Y  L  S  I  D  M  A  G  A  A  S  S  G  T  S  I  K  G  K  E  K
N_tetraspe   GCATACAGCTACCACGGCTCGGTCCCGGGGTGGAGCCATCATGGCGGTGAAGTCAGCTATCTCTCGATCGATATGGCTGGAGCTGCCAGCAGTGGAACATCGATAAAGGGAAAAGAAAAG
              A  Y  S  Y  H  G  S  V  P  G  W  S  H  H  G  G  E  V  S  Y  L  S  I  D  M  A  G  A  A  S  S  G  T  S  I  K  G  K  E  K

                370       380       390       400       410       420       430       440       450       460       470       480
             ....|....|....|....|....|....|....|....|....|....|....|....|....|....|....|....|....|....|....|....|....|....|....|....|
N_crassa     GAACTTCGGCGCCCCAGGCTCGAATCCGGCCGTGCCAGCTGGTCTTCAAGCAGGCTTGCAGCAACTGGTGGACGAATTCACTTTGAGGGGGGATCTGGCTCAGCCCCTCGAACCAGGGAC
              E  L  R  R  P  R  L  E  S  G  R  A  S  W  S  S  S  R  L  A  A  T  G  G  R  I  H  F  E  G  G  S  G  S  A  P  R  T  R  D
N_c_B_8772   GAACTTCGGCGCCCCAGGCTCGACTTCGGCCGTGCCAGCTGGTCTTCAAGCAGGCTTGCAGCAACTGGTGGACGAATTCACTTTGAAGGGGGATCTGGCTCAGCCCCTCGAACCAGGGAC
              E  L  R  R  P  R  L  D  F  G  R  A  S  W  S  S  S  R  L  A  A  T  G  G  R  I  H  F  E  G  G  S  G  S  A  P  R  T  R  D
N_c_C_8858   GAACTTCGGCGTCCCAAGCTCGACTCCGGCCGTGCCAGCTGGTCTTCAAGCAGGCTTGCAGCAACTGGTGGACGAATTCACTTCGAAGGAGGATCGGGCTCTGCCCCTCGAACCAGGGAC
              E  L  R  R  P  K  L  D  S  G  R  A  S  W  S  S  S  R  L  A  A  T  G  G  R  I  H  F  E  G  G  S  G  S  A  P  R  T  R  D
N_dis_8780   GAAATTCGGCGTCCCAGGCTCAACTCTGGCCGAGCCAGCTGGTCTTCAAGCAGGCTTTCAGCAGCTGGTGGACGATTTCACTATGAAGGAGTATCCGGCTCTGCCCTTCCAACCAGGGAC
              E  I  R  R  P  R  L  N  S  G  R  A  S  W  S  S  S  R  L  S  A  A  G  G  R  F  H  Y  E  G  V  S  G  S  A  L  P  T  R  D
N_dis_8827   GAAATTCGGCGTCCCAGGCTCAACTCTGGCCGAGCCAGCTGGTCTTCAAGCAGGCTTTCAGCAGCTGGTGGACGATTTCACTATGAAGGAGTATCCGGCTCTGCCCTTAGAACCAGGGAC
              E  I  R  R  P  R  L  N  S  G  R  A  S  W  S  S  S  R  L  S  A  A  G  G  R  F  H  Y  E  G  V  S  G  S  A  L  R  T  R  D
N_his_8815   GAACTTCGGCGTCCCAAGCTCGACTCCGGCCGTGCCAGCTGGTCTTCAAGCAGGCTTGCAGCAACTGGTGGACGAATTCACTTTGAAGGAGGATCTGGCTCTGCCCCTCGAACCAGGGAC
              E  L  R  R  P  K  L  D  S  G  R  A  S  W  S  S  S  R  L  A  A  T  G  G  R  I  H  F  E  G  G  S  G  S  A  P  R  T  R  D
N_i_A_8901   GAACTTCGGCGTCCCAGGCTCGACTCCGGCCGTGCCAGCTGGTCGTCAAGCAGGCTTGCAGCAACTGGTGGACGAATTCACTTTGAAGGAGGATCTGGCTCTGCCCCTCGAACCAGGGAC
              E  L  R  R  P  R  L  D  S  G  R  A  S  W  S  S  S  R  L  A  A  T  G  G  R  I  H  F  E  G  G  S  G  S  A  P  R  T  R  D
N_i_B_8768   GAACTTCGGCGTCCCAGGCTCGACTCCGGCCGAGCCAGCTGGTCTTCAAGCAGGCTTGCAGCAACTGGTGGACGAATTCACTTTGAAGGAGGATCTGGCTCTGCCCCTCGGACCAGAGAC
              E  L  R  R  P  R  L  D  S  G  R  A  S  W  S  S  S  R  L  A  A  T  G  G  R  I  H  F  E  G  G  S  G  S  A  P  R  T  R  D
N_met_8853   GAACTTCGGCGTCCCAGGCTCGACTCCGGCCGTGCCAGCTGGTCTTCAAGCAGGCTTGCAGCAACTGGTGGACGAATTCACTTTGAAGGAGGATCTGGCTCTGCCCCTCGAACCAGGGAC
              E  L  R  R  P  R  L  D  S  G  R  A  S  W  S  S  S  R  L  A  A  T  G  G  R  I  H  F  E  G  G  S  G  S  A  P  R  T  R  D
N_per_8835   GAACTTCGGCGTCCCAAGCTCGACTCCGGCCGTGCCAGCTGGTCTTCAAGCAGGCTTGCAGCAACTGGTGGACGAATTCACTTCGAAGGAGGATCGGGCTCTGCCCCTCGAACCAGGGAC
              E  L  R  R  P  K  L  D  S  G  R  A  S  W  S  S  S  R  L  A  A  T  G  G  R  I  H  F  E  G  G  S  G  S  A  P  R  T  R  D
N_sit_0412   GAACTTCGGCGTCCCAAGCTCGACTCCGGCCATGCCAGCTGGTCTTCAAGCAGGCTTGCAGCAACTGGTGGACGGATTCACTTTGAAGGACGATCTGGCTCTGTCCCTCGAACCAGGGAC
              E  L  R  R  P  K  L  D  S  G  H  A  S  W  S  S  S  R  L  A  A  T  G  G  R  I  H  F  E  G  R  S  G  S  V  P  R  T  R  D
N_tetraspe   GAACTTCGGCGTCCCAAGCTCGACTCCGGCCGTGCCAGCTGGTCTTCAAGCAGGCTTGCAGCAACTGGTGGACGAATTCACTTTGAAGGAGGATCTGGCTCTGCCCCTCGAACCAGGGAC
              E  L  R  R  P  K  L  D  S  G  R  A  S  W  S  S  S  R  L  A  A  T  G  G  R  I  H  F  E  G  G  S  G  S  A  P  R  T  R  D
```

```
                490       500       510       520       530       540       550       560       570       580       590       600
            ....|....|....|....|....|....|....|....|....|....|....|....|....|....|....|....|....|....|....|....|....|....|....|....|
N_crassa    GGCTCGAGTTCCCCCGTCCAGAGCTCAAGCAACTCCAGCAGCTCAGAAGACGATTTGGAGATCACTCCCGTAAAGAAATTTACCAGCGCGGTGACAACCTCAAGTCCACTCGCGGGAGTA
             G  S  S  S  P  V  Q  S  S  S  N  S  S  S  S  E  D  D  L  E  I  T  P  V  K  K  F  T  S  A  V  T  T  S  S  P  L  A  G  V
N_c_B_8772  GGCTCGAGTTCCCCCGTCCAGAGATCAAGCAACTCCAGCAGCTCAGAAGACGATTTGGAGATCACGCCTGTAAAGAAATTTACCAGCGCGGTGACAACCTCAAGTCCACTCGCGGGAGTA
             G  S  S  S  P  V  Q  R  S  S  N  S  S  S  S  E  D  D  L  E  I  T  P  V  K  K  F  T  S  A  V  T  T  S  S  P  L  A  G  V
N_c_C_8858  GGCTCGAATTCCCCCGTCCAGAGCTCCAGCAACTCCAGTAGCTCAGAAGACGATTTGGAGATCACGCCCGTGAGGAAATTTACCAGCGTAGTGACAACCTCAAGTCCACTCGCGGGAGTA
             G  S  N  S  P  V  Q  S  S  S  N  S  S  S  S  E  D  D  L  E  I  T  P  V  R  K  F  T  S  V  V  T  T  S  S  P  L  A  G  V
N_dis_8780  GGCTCGAGTTCCCCCGTCCAGAGCTCCAGCAACTCCAGCAGCTCGGAAGACGATTTGGAGATCAAGCCCGTGAAGAACTTTACCAGCGCCGTGACAATCTCCAGTCCTCTCGCAGAAGTA
             G  S  S  S  P  V  Q  S  S  S  N  S  S  S  S  E  D  D  L  E  I  K  P  V  K  N  F  T  S  A  V  T  I  S  S  P  L  A  E  V
N_dis_8827  GGCTCGAGTTCCCCCGTCCAGAGCTCCAGCAACTCCAGCAGCTCGGAAGACGATTTGGAGATCAAGCCCGTGAAGAACTTTACCAGCGCCGTGACAATCTCCAGTCCTCTCGCAGAAGTA
             G  S  S  S  P  V  Q  S  S  S  N  S  S  S  S  E  D  D  L  E  I  K  P  V  K  N  F  T  S  A  V  T  I  S  S  P  L  A  E  V
N_his_8815  GGCTCGAATTCCCCCGTCCAGAGCTCCAGCAACTCCAGCAGCTCAGAAGACGATTTGGAGATCACGCCCGTGAGGAAATTTACCAGCGCAGTGACAACCTCAAGTCCGCTCGCGGGAGTA
             G  S  N  S  P  V  Q  S  S  S  N  S  S  S  S  E  D  D  L  E  I  T  P  V  R  K  F  T  S  A  V  T  T  S  S  P  L  A  G  V
N_i_A_8901  GGCTCGAATTCCCCCGTCCAGAGCTCAAGCAACTCCACCAGCTCAGAAGATGATTTGGAGATCACGCCCGTGAGGAAATTTACCAGCGCGGTGACAACCTCAAGTCCGCTCGCGGGAGTA
             G  S  N  S  P  V  Q  S  S  S  N  S  T  S  S  E  D  D  L  E  I  T  P  V  R  K  F  T  S  A  V  T  T  S  S  P  L  A  G  V
N_i_B_8768  GGCTCGAATTCCCCCGTCCAGAGCTCCAGCAACTCCAGCAGCTCAGAAGACGATTTGGAGATCACGCCCATGAGGAAATTTACCAGCGCGGTGACAACCTCAAGTCCGCTCGCGGGAGTA
             G  S  N  S  P  V  Q  S  S  S  N  S  S  S  S  E  D  D  L  E  I  T  P  M  R  K  F  T  S  A  V  T  T  S  S  P  L  A  G  V
N_met_8853  GGCTCGAATTCCCCCGTCCAGAGCTCCAGCAACTCCAGCAGCTCCGAAGACGATTTGGAGATCACGCCCGTGAGGAAATTTACCAGCGCGGTGACAACCTCAAGTCCGCTCGCGGGAGTC
             G  S  N  S  P  V  Q  S  S  S  N  S  S  S  S  E  D  D  L  E  I  T  P  V  R  K  F  T  S  A  V  T  T  S  S  P  L  A  G  V
N_per_8835  GGCTCGAATTCCCCCGTCCAGAGCTCCAGCAACTCCAGTAGCTCAGAAGACGATTTGGAGATCACGCCCGTGAGGAAATTTACCAGCGTAGTGACAACCTCAAGTCCACTCGCGGGAGTA
             G  S  N  S  P  V  Q  S  S  S  N  S  S  S  S  E  D  D  L  E  I  T  P  V  R  K  F  T  S  V  V  T  T  S  S  P  L  A  G  V
N_sit_0412  GGCTCAAAATCCCCCGTCCAGAGCTCCAGCAGCTCCAGCAGCTCAGAAGACGATTTGGAGATCACGCCCGTGGGGAAATTTACCAGCGCAGCGACAACCTCAAGTCCGCTCGCAGGAGTA
             G  S  K  S  P  V  Q  S  S  S  S  S  S  S  S  E  D  D  L  E  I  T  P  V  G  K  F  T  S  A  A  T  T  S  S  P  L  A  G  V
N_tetraspe  AGCTCGAATTCCCCCGTCCAGAGCTCCAGCAACTCCAGCAGCTCAGAAGACGATTTGGAGATCACGCCCGTGAGGAAATTTACCAGCGCAGTGACAACCTCAAGTCCGCTCGCGGGACTA
             S  S  N  S  P  V  Q  S  S  S  N  S  S  S  S  E  D  D  L  E  I  T  P  V  R  K  F  T  S  A  V  T  T  S  S  P  L  A  G  L

                610       620       630       640       650       660       670       680       690       700       710       720
            ....|....|....|....|....|....|....|....|....|....|....|....|....|....|....|....|....|....|....|....|....|....|....|....|
N_crassa    CCCAGCCGGCCGACAAGCGACGCCGACTCTGTTCACCGTCTTCATCCCGGTCATGCCCGAAAAGTGTCAAACTCATCATACAAACAAGCCGGACAGTCCTCATCGCCCAGTCAGTCATCC
             P  S  R  P  T  S  D  A  D  S  V  H  R  L  H  P  G  H  A  R  K  V  S  N  S  S  Y  K  Q  A  G  Q  S  S  S  P  S  Q  S  S
N_c_B_8772  CCCAGCCGGCCGACAAGCGACGCCGACTCTGTTCACCGTCTTCATCCCGGTCATGCCCGAAAAGTGTCAAACTCA---TACAAACAAGCCGGACAGTCCTCATCGCCCAGTCAGTCATCC
             P  S  R  P  T  S  D  A  D  S  V  H  R  L  H  P  G  H  A  R  K  V  S  N  S     Y  K  Q  A  G  Q  S  S  S  P  S  Q  S  S
N_c_C_8858  CCCAGTCGGCCGACAAGCGACACCGACTCTGTTCACCGTCTTCATCCC---------CGAAAAGTGTCAAACTCATCCTACAAACAAGCCGGACAGTCCTCATCGCCCAGTCAGTCATCC
             P  S  R  P  T  S  D  T  D  S  V  H  R  L  H  P           R  K  V  S  N  S  S  Y  K  Q  A  G  Q  S  S  S  P  S  Q  S  S
N_dis_8780  TCGAGTCGGCCGACAAGCGATACCGACTCCGTTCACCGTCTTCATCCTGGTCATGCCCGAAATATGTCAAACTCATCAAACAAACAAGACGGACCATCCTCATCGCCCAGTCAGTTATTC
             S  S  R  P  T  S  D  T  D  S  V  H  R  L  H  P  G  H  A  R  N  M  S  N  S  S  N  K  Q  D  G  P  S  S  S  P  S  Q  L  F
N_dis_8827  TCGAGTCGGCCGATAAGCGATACCGACTCCGTTCACCGTCTTCATCCTGGTCATGCCCGAAATATGTCAAACTCATCAAACAAACAAGACGGACCATCCTCATCGCCCAGTCAGTTATTC
             S  S  R  P  I  S  D  T  D  S  V  H  R  L  H  P  G  H  A  R  N  M  S  N  S  S  N  K  Q  D  G  P  S  S  S  P  S  Q  L  F
N_his_8815  CCCAGCCGGCGGGCAAGCGACACCGACTCTGTTCACCGTCTCCATCCCGGTCATGCCCGAAATGTGTCAAACTCATCATACAAACAAGCCGGACAATCCTCATCGCCCAGTCAGTCATCC
             P  S  R  R  A  S  D  T  D  S  V  H  R  L  H  P  G  H  A  R  N  V  S  N  S  S  Y  K  Q  A  G  Q  S  S  S  P  S  Q  S  S
N_i_A_8901  CCCAGCCGGCCGACAAGCGACACCGACTCTGTTCACCGTCTTCATCCCGGCCATGCCCGAAATGTGTCAAACTCATCATACAAACAAGCCGGGCAATCCGCGTCGCCCAGTCAGTCATCC
             P  S  R  P  T  S  D  T  D  S  V  H  R  L  H  P  G  H  A  R  N  V  S  N  S  S  Y  K  Q  A  G  Q  S  A  S  P  S  Q  S  S
N_i_B_8768  CCCAGTCGGCCGACAAGCGACACCGACTCTGTTCACCGTCTTCATCCCGGCCATGCCCGAAATGTGTCAAACTCATCATACAAACAAGCCGGACAATCCGCGTCGCCCAGTCAGTCATCC
             P  S  R  P  T  S  D  T  D  S  V  H  R  L  H  P  G  H  A  R  N  V  S  N  S  S  Y  K  Q  A  G  Q  S  A  S  P  S  Q  S  S
N_met_8853  CCCAGCCGGCCGACAAGCGACACCGACTCTGTTCACCGTCTCCATCCCGGTCATGGCCGAAATGTATCAAACTCATCATACAAACAAGCCGGACAATCCGCGTCGCCCAGTCAGTCATCC
             P  S  R  P  T  S  D  T  D  S  V  H  R  L  H  P  G  H  G  R  N  V  S  N  S  S  Y  K  Q  A  G  Q  S  A  S  P  S  Q  S  S
N_per_8835  CCCAGTCGGCCGACAAGCGACACCGACTCTGTTCACCGTCTTCATCCCGGTCATGCCCGAAAAGTGTCAAACTCATCATACAAACAAGCCGGACAATCCTCATCGCCCAGTCAGTCATCC
             P  S  R  P  T  S  D  T  D  S  V  H  R  L  H  P  G  H  A  R  K  V  S  N  S  S  Y  K  Q  A  G  Q  S  S  S  P  S  Q  S  S
N_sit_0412  CCCAGCCGGCGGACAAGCGACACCGACTCTGTTCACCGTTTCCATCCCGGTCATGCCCGAAATGTGTCAAACTCATCATACAAACAAGCCGGACAATCCTCATCGCCCAGTCAGTCATCC
             P  S  R  R  T  S  D  T  D  S  V  H  R  F  H  P  G  H  A  R  N  V  S  N  S  S  Y  K  Q  A  G  Q  S  S  S  P  S  Q  S  S
N_tetraspe  CCCAGCCGGCGGACAAGCGACACCGACTCTGTTCACCGTCTCCATCCCGGTCATGCCCGAAATGTGTCAAACTCATCATACAAACAAGCCGGACATTCCTCATCGCCCAGTCAGTCATCC
             P  S  R  R  T  S  D  T  D  S  V  H  R  L  H  P  G  H  A  R  N  V  S  N  S  S  Y  K  Q  A  G  H  S  S  S  P  S  Q  S  S
```

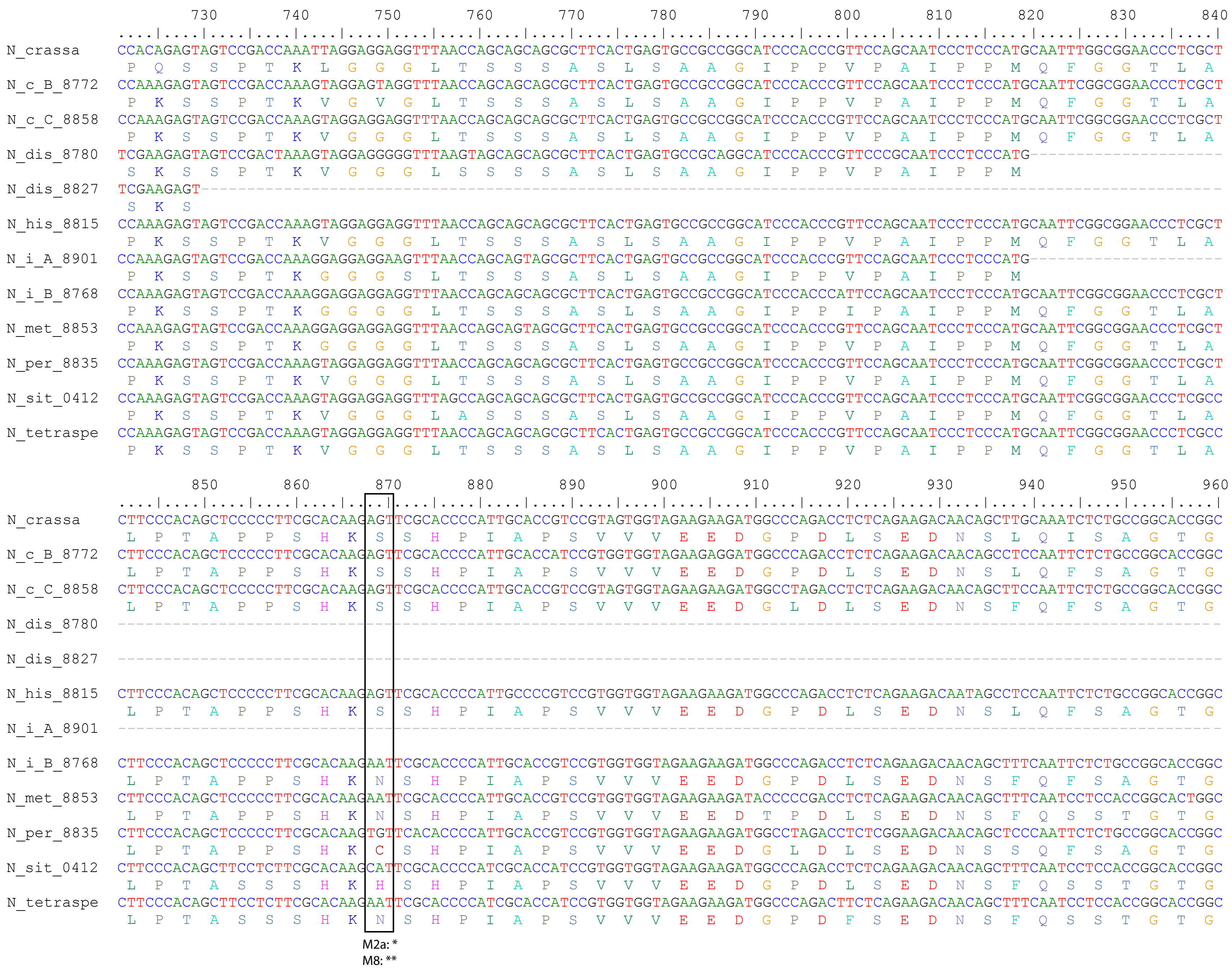
N_crassa
N_c_B_8772
N_c_C_8858
N_dis_8780
N_dis_8827
N_his_8815
N_i_A_8901
N_i_B_8768
N_met_8853
N_per_8835
N_sit_0412
N_tetraspe
M2a: *
M8: **

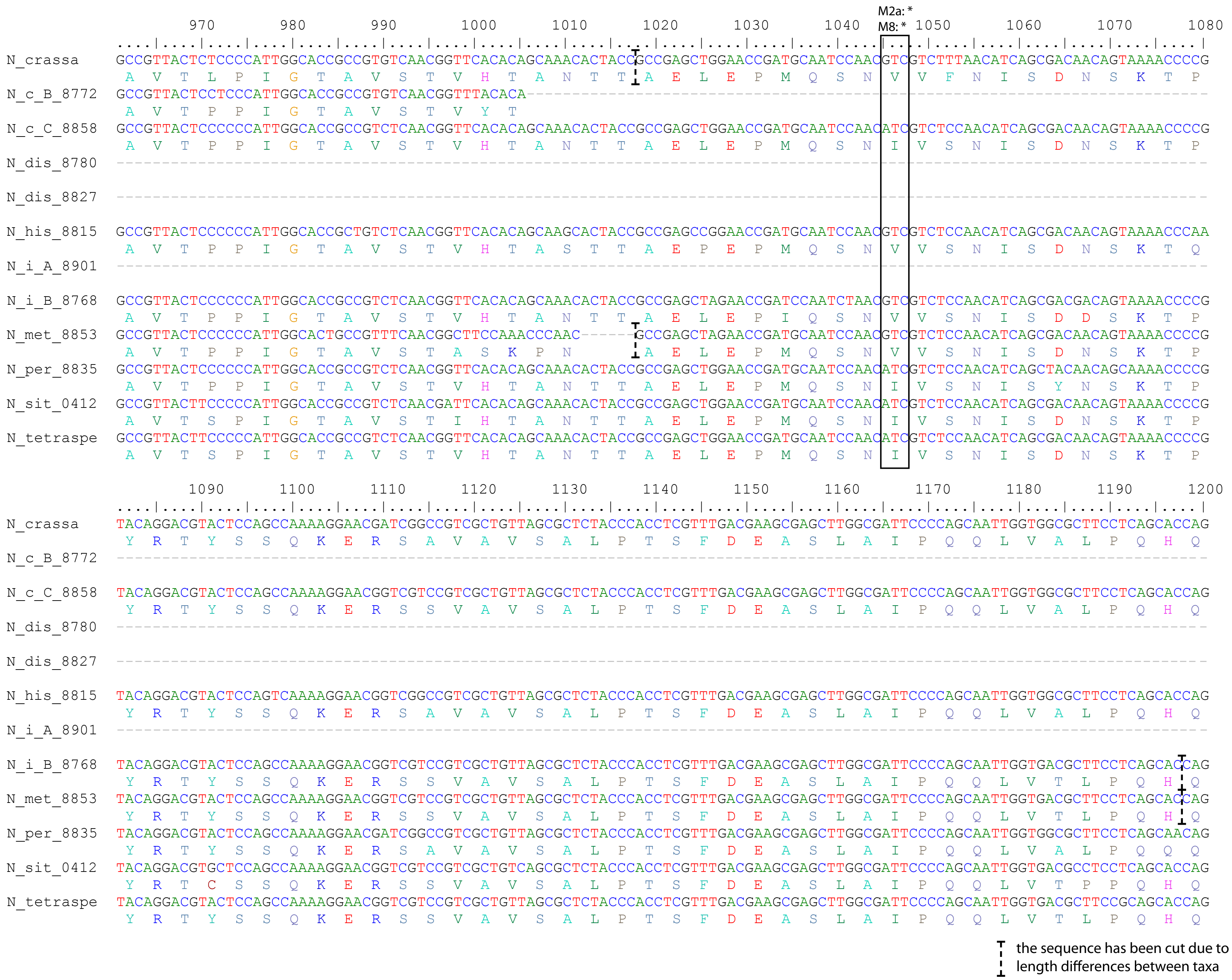

M2a: *
M8: *
970 980 990 1000 1010 1020 1030 1040 1050 1060 1070 1080
1090 1100 1110 1120 1130 1140 1150 1160 1170 1180 1190 1200
N_crassa
N_c_B_8772
N_c_C_8858
N_dis_8780
N_dis_8827
N_his_8815
N_i_A_8901
N_i_B_8768
N_met_8853
N_per_8835
N_sit_0412
N_tetraspe
the sequence has been cut due to length differences between taxa

```
                     1210      1220
              ....|....|....|....|.
N_crassa      ACAACTGGGAAGTTGGGGAAA
               T  T  G  K  L  G  K
N_c_B_8772    ---------------------

N_c_C_8858    GCAACTGGGAAG---------
               A  T  G  K
N_dis_8780    ---------------------

N_dis_8827    ---------------------

N_his_8815    GCAACTGGGAAGTTGGGGAAA
               A  T  G  K  L  G  K
N_i_A_8901    ---------------------

N_i_B_8768    GCAACTGGGAAGTTGGGGAAA
               A  T  G  K  L  G  K
N_met_8853    GCAACTGGGAAG---------
               A  T  G  K
N_per_8835    ACAAGTGGAAAG---------
               T  S  G  K
N_sit_0412    GCAACTGGGAAGTTGGGGAAA
               A  T  G  K  L  G  K
N_tetraspe    GCAACTGGGAAGTTGGGGAAA
               A  T  G  K  L  G  K
```

Gene: NCU06387
Compared to the annotated sequence for N. crassa, the alignment is missing the first 37 codons and the last 36 codons.
The sequence N_crassa is the annotated gene sequence for NCU06387.
The sequence N_tetraspe is the gene sequence from the N. tetrasperma genome sequence.

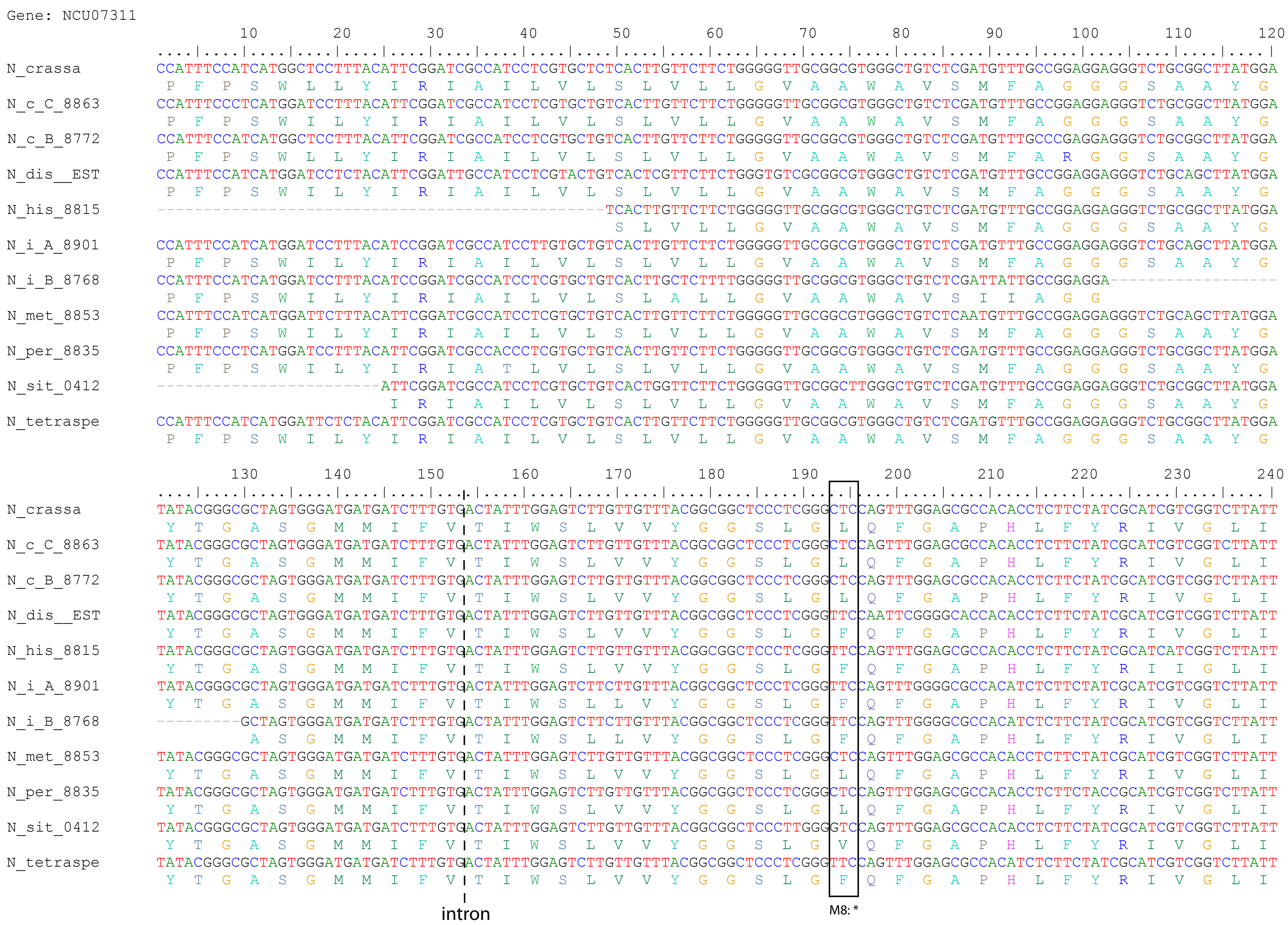

Gene: NCU07311
N_crassa
N_c_C_8863
N_c_B_8772
N_dis__EST
N_his_8815
N_i_A_8901
N_i_B_8768
N_met_8853
N_per_8835
N_sit_0412
N_tetraspe
intron
M8: *

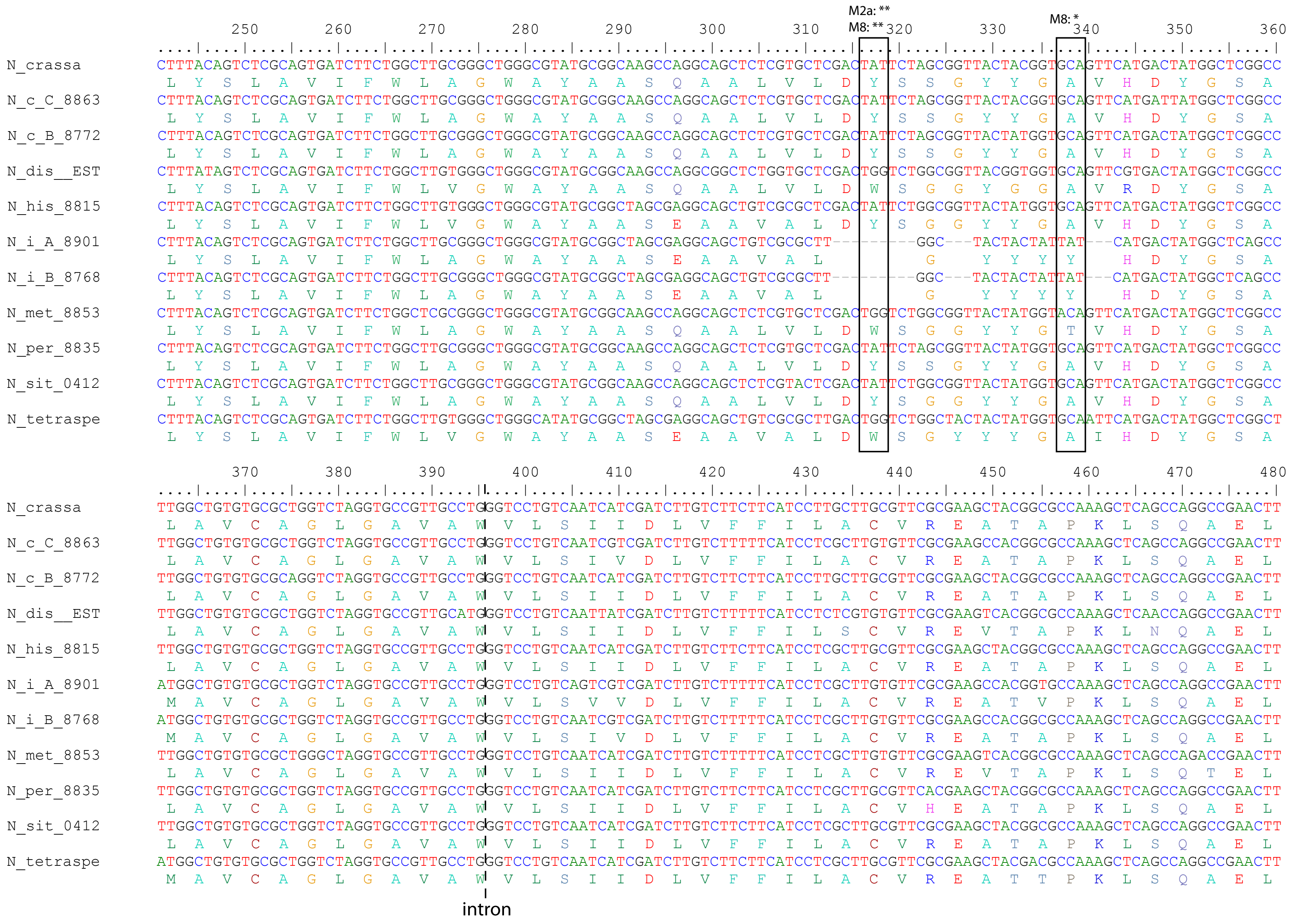

M2a: **
M8: **
M8: *
N_crassa
N_c_C_8863
N_c_B_8772
N_dis__EST
N_his_8815
N_i_A_8901
N_i_B_8768
N_met_8853
N_per_8835
N_sit_0412
N_tetraspe
intron

```
                        490       500       510       520
                ....|....|....|....|....|....|....|....|..
N_crassa        GGACAAGTACAGCCAACCGCCTCCGTTGCGCCCGCCACTGCT
                 G  Q  V  Q  P  T  A  S  V  A  P  A  T  A
N_c_C_8863      GGACAAGTACAACCAACCGCCTCCGTT---------------
                 G  Q  V  Q  P  T  A  S  V
N_c_B_8772      GGACAAGTACAACCAACCGCCTCCGTTGCGCCCGCCACTGCT
                 G  Q  V  Q  P  T  A  S  V  A  P  A  T  A
N_dis__EST      GGACAAGTACAGCCAACCGCCTCTGTTGCGCCCGGCACTGCT
                 G  Q  V  Q  P  T  A  S  V  A  P  G  T  A
N_his_8815      GGACAAGTA---------------------------------
                 G  Q  V
N_i_A_8901      GGACAAGTACAGCCAACCGCCTCCGTTACGCCCGCCACT---
                 G  Q  V  Q  P  T  A  S  V  T  P  A  T
N_i_B_8768      GGACAAGTACAGCCAACCGCCTCCGTTACGCCCGCCACTGCT
                 G  Q  V  Q  P  T  A  S  V  T  P  A  T  A
N_met_8853      GGACAAATACAGCCAACCGCCTCCGTTGCGCCCACCACTGCT
                 G  Q  I  Q  P  T  A  S  V  A  P  T  T  A
N_per_8835      GGACAAGTACAACCAACCGCCTCCGTTACGCCCACCACTGCT
                 G  Q  V  Q  P  T  A  S  V  T  P  T  T  A
N_sit_0412      GGACAAGTACAGCCAACCGCCTCC------------------
                 G  Q  V  Q  P  T  A  S
N_tetraspe      GGACAAGTACAGCCAACCGCCTCCGTTGCGCCCGCCACTGCT
                 G  Q  V  Q  P  T  A  S  V  A  P  A  T  A
```

Gene: NCU07311
Compared to the annotated sequence for N. crassa, the alignment is missing the first 13 codons and the last 17 codons.
The sequence N_crassa is the annotated gene sequence for NCU07311.
The sequence N_tetraspe is the gene sequence from the N. tetrasperma genome sequence.
