# Supplementary material for: Analyses of expressed sequence tags in Neurospora reveal rapid evolution of genes associated with the early stages of sexual reproduction in fungi
Source: BMC Evol Biol. 2012 Nov 27;12:229. doi: 10.1186/1471-2148-12-229 (PMC3571971; doi:10.1186/1471-2148-12-229)
Supplement: Additional file 4: Table S3 — Primers used for PCR amplification of candidate genes for test for positive selection. (PDF 34 kb) [file 1471-2148-12-229-S4.pdf]

Supplementary Table 3. Primers used for PCR amplification of candidate genes for test for positive selection.

Gene	F-primer (5'...3')	R-primer (5'...3')
NCU01720	ATCACGTTGGCAAGAGGGACAT	ACCCTCCGCAACCATGAAGTAG
NCU03013	TGCGTACCTCAGTAGCTCTTTCAC	CTGCAACAGGTAGTGTGCCTGAC
NCU03584	TCCGAGTCCAATGTCACCACTC	CAGGTACTCATCCCACCTGTTG
NCU06387	GCAAGCAGCAAAGGAACAGAAC	AAGCTCCATCGACTCTTCTTCG
NCU07311	CTGTTGGAGTCCCTCCATTTCC	GGAGGAAGCTCATTAGCAGTGG
